# Optimization of industrial (3000 L) production of *Bacillus subtilis* CW-S and its novel application for minituber and industrial-grade potato cultivation

**DOI:** 10.1038/s41598-022-15366-5

**Published:** 2022-07-01

**Authors:** Md. Abuhena, Jubair Al-Rashid, Md. Faisal Azim, Md. Niuz Morshed Khan, Md. Golam Kabir, Nirmal Chandra Barman, Noorain Munim Rasul, Shahina Akter, Md. Amdadul Huq

**Affiliations:** 1 Department of Research and Development, Apex Biofertilizers and Biopesticides Limited, Gobindaganj, Gaibandha, 5740 Bangladesh; 2 Apex Biotechnology Laboratory, Apex Holdings Ltd., East Chandora, Shafipur, Kaliakoir, Gazipur, 1751 Bangladesh; 3 Department of Food Science and Biotechnology, College of BioNano Technology, Gachon University, Seongnam, 461-701 Republic of Korea; 4 Department of Food and Nutrition, College of Biotechnology and Natural Resource, Chung-Ang University, Anseong, Gyeonggi-do 17546 Republic of Korea

**Keywords:** Microbiology, Industrial microbiology

## Abstract

A commercial plant probiotic product was developed employing *Bacillus subtilis* CW-S in submerged fermentation. The effects of molasses and urea on cell growth were investigated with the goal of low-cost manufacturing. Plackett–Burman and Central-Composite Design (CCD) were utilized to optimize production parameters to maximize productivity. The stability of the formulated product and its efficacy in cultivating minituber in aeroponics and industrial-grade potatoes in the field were assessed. The results showed that the medium BS10 (molasses and urea) produced satisfactory cell density (7.19 × 10^8^ CFU/mL) as compared to the control (1.51 × 10^7^ CFU/mL) and BS1-BS9 (expensive) media (1.84 × 10^7^–1.37 × 10^9^ CFU/mL). According to validated CCD results, optimized parameters fitted well in pilot (300 L; 2.05 × 10^9^ CFU/mL) and industrial (3000 L; 2.01 × 10^9^ CFU/mL) bioreactors, resulting in a two-fold increase in cell concentration over laboratory (9.84 × 10^8^ CFU/mL) bioreactors. In aeroponics, CW-S produced excellent results, with a significant increase in the quantity and weight of minitubers and the survival rate of transplanted plantlets. In a field test, the yield of industrial-grade (> 55 mm) potatoes was increased with a reduction in fertilizer dose. Overall, the findings suggest that CW-S can be produced commercially utilizing the newly developed media and optimized conditions, making plant probiotics more cost-effective and accessible to farmers for crop cultivation, particularly in aeroponic minituber and industrial-grade potato production.

## Introduction

The term "plant probiotic bacteria" was first coined by Haas and Keel for bacteria that possess three key characteristics that contribute to better plant protection: (i) the ability to initiate induced systemic resistance (ISR) in their hosts, (ii) effectiveness and competitiveness in niche colonization, and (iii) the presence of direct antagonistic traits on pathogens^1^. Plant probiotics are microbial cultures that promote plant growth and/or biocontrol through a variety of activities such as phosphate solubilization, nitrogen fixation, siderophores production, and enhanced plant immunity against various phytopathogens-caused diseases^2,3^. *Bacillus subtilis*, as a plant probiotic, improves nutrient solubilization, accelerates nutrient absorption in soil, and acts as a plant growth-promoting bacteria (PGPB). It can also fix nitrogen by expressing the nifH gene^4^. It is a nonpathogenic bacteria that is widely used as a model organism in secondary metabolite production. The Food and Drug Administration (FDA) has classified *B. subtilis*, along with other *Bacillus* species, as a GRAS (Generally Recognized As Safe) organism. *Bacillus subtilis* improves soil structure by combining soil particles together through extracellular metabolite secretion, enhances the breakdown of complex organic material as well as insoluble nutrients into simpler forms that can be absorbed easily by plants, improves their growth, and induces resistance to stress and disease^2,5^.

Recently, there has been a lot of interest in plant probiotics. Some *Bacillus *spp*.* have the potential to be plant probiotics and can be grown on a large scale^6^. Poor soil conditions, adverse climatic situations, extreme temperatures and drought, traditional farming practices, and lack of technological development have significantly affected crop productivity in the northern region of Bangladesh^7^. In recent years, the emphasis on green farming and high-value crop production has led to a transformation in agrochemical inputs^8^. The search for economical and ecologically safe solutions that enhance the growth and yield of plants has led to the use of biofertilizers as an alternative to agrochemicals^9^. In developed countries, *bacillus*-based products are widely available. Alinit, the first commercially available bacterial fertilizer, contains *Bacillus *spp. It resulted in a 40% increase in crop output. *Bacillus *spp.-based crop improvement products include Kodiak (*Bacillus subtilis GB03*), Quantum-400 (*Bacillus subtilis GB03*), and YIB (*Bacillus subtilis QST713*). *Bacillus*-based biofertilizers are more active than other bacterial fertilizers because *Bacillus *spp. in commercial formulations have higher cell viability due to more efficient metabolite production and spore-forming abilities^10^.

The manufacturing cost of microbial products will be a critical factor if they are to be encouraged in less developed countries because of a shortage of raw material resources and an optimized industrial production method. The cell concentration of each native strain of *Bacillus *spp. is different. It is due to their nutritional requirements and cultural conditions. A practical way to cut manufacturing costs is to optimize culture time, use inexpensive media, and optimize the production parameters to obtain a higher cell concentration. The most common and efficient method of getting significant amounts of biomass is through low-cost products and by-products. By minimizing industrial waste and giving these products a second life and value, this waste-based microorganism growth media design will contribute to the bio-economy^11^.The ingredients of a medium must contain the basic requirements for cell growth and also provide adequate energy for metabolism and cell survival. In the majority of cases, nitrogen and carbon supplies are essential. Nitrogen sources can be inorganic (as in ammonium and nitrate salts) or organic (such as amino acids, proteins, or urea). Sugars like sucrose and fructose, as well as industrial byproducts like molasses, are common carbon sources^12^. Industrial production incurs considerable operating costs, which consequently increases the product price^13,14^.

Statistical research methods such as Plackett–Burman and response surface methodology (RSM) can optimize all influencing parameters at a time, circumventing the constraints of a single-factor optimization procedure^15^. Plackett–Burman design (PBD) is a rapid and convenient approach to finding the critical components, saving time and ensuring that each parameter is well-defined^16^. RSM is used to explore the influence of several factors that affect responses by modifying them simultaneously and executing a series of experiments using CCD^17^. PBD and RSM have effectively optimized some bioprocesses^18,19^. In recent times, different researchers have published statistically optimized process designs of *B. subtilis* for the production of various enzymes and metabolites^20,21^. However, industrial production data on *Bacillus subtilis* as a plant probiotic is still limited.

Bangladesh is the world's seventh-largest producer of potatoes. It has a total yearly production of nearly 10.21 million tons^22^.Potatoes are a popular high-consumption food because they are abundant in carbohydrates, fat, fiber, and important micronutrients including potassium, salt, and carotenoids^23^. People consume potatoes in different ways: boiled or fried, in processed foods such as chips, French fries, powder, and potato dal. Potatoes are graded according to their intended use. Smaller-sized potatoes are used as seeds, and larger sizes are used in the industry. Seed potatoes must be free of viruses, have large eyes, and have high yield potential, and industrial potatoes must have a high relative density and low levels of reducing sugar and phenol. Many studies have shown that different parameters like soil type, fertilizer application, dates of planting and harvesting, and genetic factors influence the production of potato tubers and their external and internal properties^24^. Field farming faces risks and uncertainties, both biotic and abiotic, such as high winds, floods, droughts, and pest infestations. The aeroponic system is now widely used to grow minituber for potato seed production. Plant roots in an aeroponic system have better access to ambient levels of gases like oxygen, which allow them to grow faster and more predictably. These enclosed systems demand less irrigation than soil-grown plants. The nutrients can be recycled because they are dissolved in the water. Apart from these benefits, the ability to produce a large number of plants in small spaces also contributes to the environmental friendliness of aeroponics^25^.

In this work, we focused on the low-cost industrial production of a very effective plant probiotic inoculant for farmers developed from the native strain *Bacillus subtilis* CW-S, as well as the product's novel applications, such as aeroponic minituber and industrial-grade potato production.

## Results

### Morphological, biochemical, and molecular identification of CW-S

CW-S cells were rod-shaped and produced oval endospores. They were gram-positive and gave a positive VP test but a negative MR test. CW-S was also positive for starch hydrolysis, catalase, and citrate activity, as well as grown in NA media containing 7.0% NaCl at 45–50 °C. They were able to ferment mannitol, which was indicated by the development of a yellow hue in *Bacillus* differentiation agar. The isolate CW-S was putatively identified as *B. subtilis.* The biochemical tests, colony morphology, and microscopic observations are summarized in Supplementary Table T1 and Supplementary Fig. F1, respectively. DNA similarity was determined using the NCBI BLAST service (http://www.ncbi.nlm.nih.gov). The sequence had 100% query coverage and 100% identity with the *Bacillus subtilis* strain DK15 16S ribosomal RNA gene, partial sequence, and complete sequence. The NCBI reference sequence was MT534533.1. The sequence number MW819960.1 was assigned to the sequence when it was deposited into the GenBank database. The phylogenetic tree showed (Supplementary Fig. F2) that the typical *Bacillus subtilis* strain sequence in the current investigation was in the same clade as *Bacillus subtilis* sequences from the gene bank, validating the strain identification.

### Plant probiotic potential in *Bacillus subtilis* CW-S

The potential of CW-S to fix nitrogen from the environment was confirmed by growth in the Nfb medium, as shown in Supplementary Fig. F3. In a quantitative analysis, CW-S showed P solubilization activity (10.22 ppm) and produced IAA (05.342 g/mL) in a nutritional broth medium supplemented with tryptophan.

### Antagonistic activity of CW-S (*Bacillus subtilis*)

CW-S inhibited phytopathogens efficiently on PDA media. CW-S had the most potent antagonistic activity against *Curvularia spicifera* ApexBLR4 (69.9 ± 3.81%) and *Alternaria alternata* ApexALT10 (68.1 ± 4.71). The lowest inhibition was observed against *Lasiodiplodia theobromae* ApexRHZ5K18 (51.6 ± 4.24) and *Fusarium equiseti* ApexPTR3 (53.7 ± 3.67), while moderate inhibition was detected against *Sclerotium delphinii* Apex_SCR5 (63.5 ± 4.04).

### PGPR-seedling assay of CW-S

Table 1 compares the effects of the CW-S strain (T_1_) on wheat seedlings with the control (T_0_). According to the data, all vegetative growth parameters were higher in treated seedlings than in control seedlings. The inoculants had no negative effect on the seedlings' leaf number, tiller number, leaf diameter, shoot height, root length, and fresh and dry weight. Seedling inoculation resulted in an increase of 18.33% in leaves, 50% in tillers, 20.06% in leaf diameter, 9.10% in plant height, 54.114% in root length, and 61.83% in fresh weight. Consequently, the dry weight of wheat seedlings increased by 55.0% compared to the control. Finally, it was concluded that the CW-S was found to have growth-promoting effects on plants.

**Table 1:** The effects of the CW-S strain on wheat seedlings in compared to control.

Treatment	Number of leaf/plant	Number of tillers/ plant	Leaf diameter (mm)	Plant height (cm)/ shoot length	Root length (cm)	Fresh weight (mg/plant)	Dry weight (mg/plant)
T_1_ = T_0_ + CW-S	7.1 ± 0.699	1.5 ± 0.527	8.5 ± 1.41	9.23 ± 0.826	27.9 ± 7.78	2387 ± 502	341 ± 68.4
T_0_ = [half-strength Hoagland (-P)]	6 ± 0.333	1 ± 0.00	7.08 ± 0.624	8.46 ± 0.509	18.1 ± 2.30	1475 ± 207	220 ± 17.9
*Significance	***	**	**	*	**	***	***

*Within column, mean ± SD values. Significance symbol (*P* < 0.05), according to Tukey multiple comparisons of means test (n = 10).

Variables of significance (*p < 0.05, **p < 0.01, ***p < 0.001).

### Medium selection for submerged culture in bioreactors

**Figure 1:**
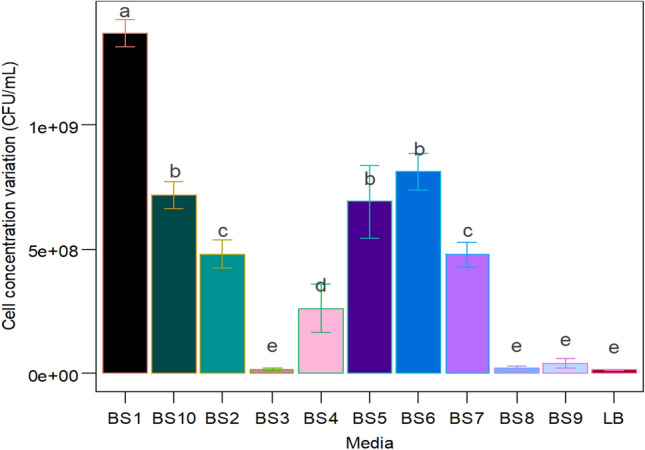
Media evaluation based on the cell concentration of CW-S.

Among the eleven different media utilized to cultivate *Bacillus subtilis* CW-S in submerged culture, BS1 yielded the highest cell concentration. The second highest cell concentrations were obtained in the BS6, BS10, and BS5, all of which were almost comparable. Figure 1 depicts the results of the growth media evaluation. In all subsequent studies, CW-S was grown on a BS10 medium containing molasses and urea since cell concentration and plant assay findings were satisfactory.

### Estimation of the total carbohydrate content of molasses samples

The expected range of total carbohydrate content of molasses is 50 ± 2%^26^. This range meets the industrial standard for the economical production of *Bacillus subtilis.* The percentage of total carbohydrates in the collected molasses sample was found to be 57 mg/mL, i.e., 57% (m/m) on average. This percentage was very suitable for *Bacillus subtilis* CW-S production.

### Identification of significant factors using PBD

**Figure 2:**
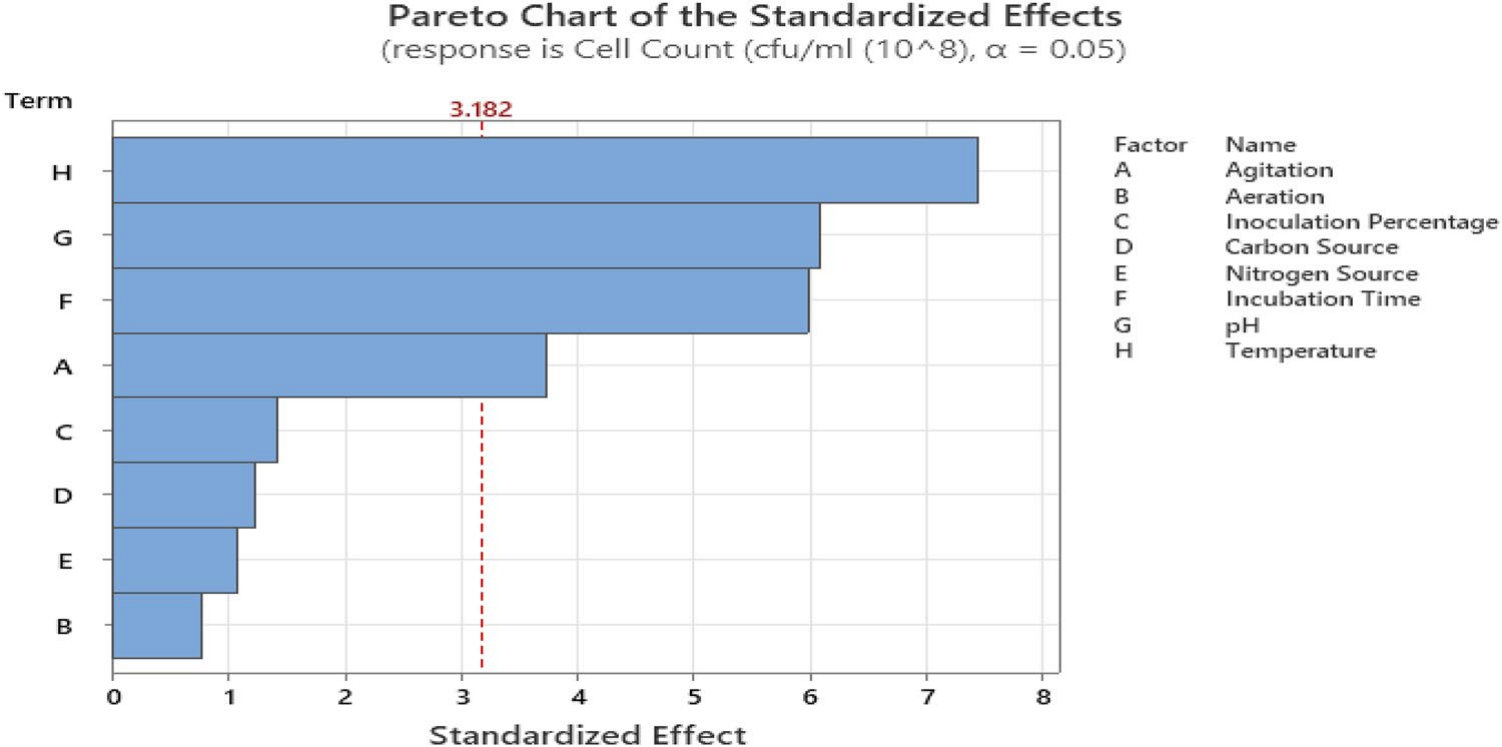
Pareto chart of the standardized effects for cell concentration of CW–S.

As shown in Table 2, there was a substantial variation in the cell concentration of strain CW-S in PBD. The concentration of cells ranged from 2.8 × 10^8^ to 7.68 × 10^8^ (CFU/mL). The variation indicates that the process must be optimized to increase the cell concentration of strain CW-S. Four factors, namely temperature, pH, incubation time, and agitation, were identified as the most potent in influencing the cell concentration, with corresponding p values of less than 0.005, implying that they were significant. The remaining factors, namely aeration, inoculum size, carbon source percentage, and nitrogen source percentage, displayed *p* values (0.1), above the significance value, implying that they were not significant. Table 3 shows the main effects of the independent factors and the analysis of variance (ANOVA), regression coefficient, F values, and P values for the factors investigated in this study. With a coefficient of determination (R^2^) of 98%, it was possible to estimate cell concentration variability of up to 98%. The predetermined R^2^ (0.98) was fairly close to the adjusted R^2^ (0.9267). The model's F-value of 18.40 for cell concentration suggests that it was significant. The probability value was used to estimate the importance of each independent factor; thus, a p-value of 0.018 showed that the model was significant. Pareto plots of the standardized effects for responses were shown in Fig. 2. Temperature, pH, incubation duration, and agitation were identified as critical independent factors by ANOVA results and were considered for further investigation by CCD. As a function of independent factors, the first-order polynomial Eq. (1) expressing the cell concentration of *B. subtilis* CW-S was derived as follows:

1 $$ \begin{aligned} {\text{Y}} = & {4}.{588 } - 0.{488} \times {\text{A }} - 0.{1}0{2} \times {\text{B }} + \, 0.{185} \times {\text{C }} - \, 0.{162} \times {\text{D }} + \, 0.{142} \times {\text{E }} \\ & + \, 0.{782} \times {\text{F }} + \, 0.{785} \times {\text{G }} + \, 0.{972} \times {\text{H}} \\ \end{aligned} $$

**Table 2:** PBD experimental runs for screening the significant independent factors affecting the growth of CW-S.

Run Order	A	B	C	D	E	F	G	H	Response
	Agitation	Aeration	Inoculum Size	Carbon Source percentage	Nitrogen Source percentage	Incubation Time	pH	Temp	Cell concentration (× 10^8^ CFU/mL)
1	− 1	− 1	− 1	− 1	− 1	− 1	− 1	− 1	2.54 ± 0.09
2	1	1	− 1	1	1	− 1	1	− 1	2.94 ± 0.03
3	1	− 1	− 1	− 1	1	1	1	− 1	4.82 ± 0.04
4	− 1	− 1	− 1	1	1	1	− 1	1	5.64 ± 0.07
5	1	1	− 1	1	− 1	− 1	− 1	1	2.80 ± 0.04
6	1	− 1	1	− 1	− 1	− 1	1	1	5.10 ± 0.04
7	− 1	1	1	1	− 1	1	1	− 1	5.14 ± 0.02
8	− 1	− 1	1	1	1	− 1	1	1	6.62 ± 0.05
9	− 1	1	− 1	− 1	− 1	1	1	1	7.68 ± 0.07
10	1	− 1	1	1	− 1	1	− 1	− 1	3.42 ± 0.06
11	− 1	1	1	− 1	1	− 1	− 1	− 1	2.84 ± 0.03
12	1	1	1	− 1	1	1	− 1	1	5.52 ± 0.17

Within columns, CFU ± SD values (n = 3).

where Y is the response (cell concentration, CFU/mL); A, B, C, D, E, F, G, and H are the independent factors of agitation, aeration, inoculum size, carbon source, nitrogen source, incubation time, pH, and temperature with their respective coefficients.

**Table 3:** ANOVA for PBD.

Source	DF	Adj SS	Adj MS	*F* Value	*P* Value
Model	8	30.1968	3.7746	18.40	0.018
Linear	8	30.1968	3.7746	18.40	0.018
Agitation	1	2.8616	2.8616	13.95	0.033
Aeration	1	0.1240	0.1240	0.60	0.494
Inoculation %	1	0.4107	0.4107	2.00	0.252
Carbon Source	1	0.3136	0.3136	1.53	0.304
Nitrogen Source	1	0.2408	0.2408	1.17	0.358
Incubation Time	1	7.3320	7.3320	35.73	0.009
pH	1	7.5843	7.5843	36.96	0.009
Temperature	1	11.3296	11.3296	55.22	0.005
Error	3	0.6156	0.2052		
Total	11	30.8124	R^2^ = 98.00%, R^2^(adj) = 92.67%, R^2^(pred) = 68.04%

### Optimization of significant parameters using RSM

RSM with CCD was used to investigate the interactions between four critical variables. The real and coded values used in the CCD are detailed in Table 7. The CCD was used in the experimental trials, as illustrated in Supplemental Table T2**.** According to the model summary (Supplementary Table T3), the linear, 2FI, and cubic models were not appropriate for this response. The adjusted R^2^, predicted R^2^ values, and lack of fit tests also demonstrated that all models except quadratic were not fit (Supplementary Table T4) for this response. As a result, the quadratic model was used to characterize the relationship between cell concentration and independent variables. The second-order polynomial Eq. (2) was obtained using multiple regression analysis:

2 $$ \begin{aligned} {\text{Y }} = & + {9}.{78} - 0.{1479} \times {\text{A }} + \, 0.{1}0{21} \times {\text{B}} - 0.{3279} \times {\text{C}} - 0.0{646} \times {\text{D }} + \, 0.0{319} \times {\text{AB}} - 0.0{881} \times {\text{AC}} \\ & + \, 0.{2819} \times {\text{AD }} + \, 0.{1256} \times {\text{BC}} - 0.{2}0{44} \times {\text{BD }} + \, 0.{12}0{6} \times {\text{CD}} - {1}.{58} \times {\text{A}} - 0.{27}0{1} \times {\text{B}} \\ & - 0.{49}0{1} \times {\text{C}}^{{2}} - 0.{9976} \times {\text{D}} \\ \end{aligned} $$

where Y stands for cell concentration response, while A, B, C, and D stand for temperature, incubation time, pH, and agitation coded values, respectively.

**Figure 3:**
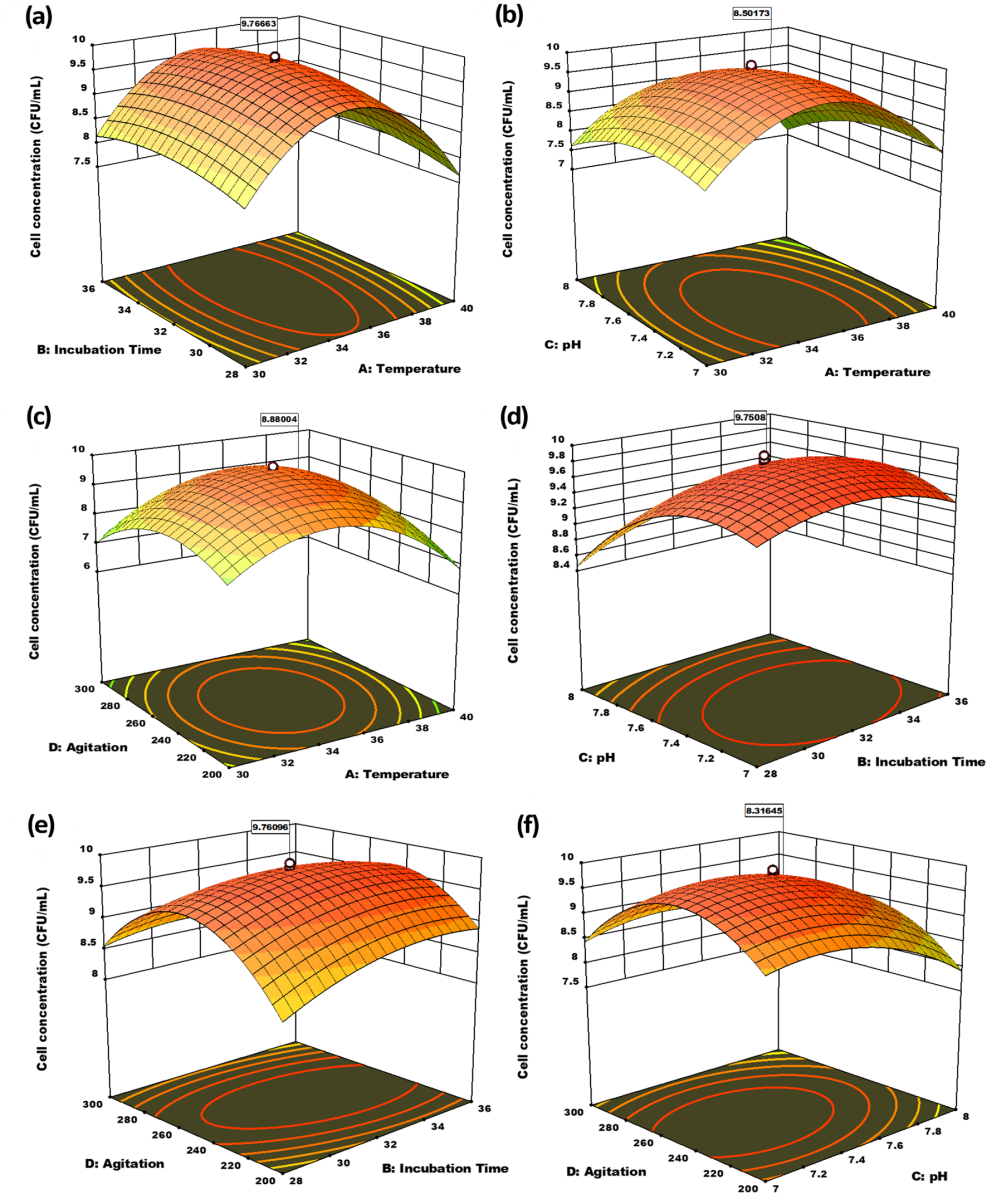
3D response surface displaying the interaction of all those components considered in CCD optimization. (**a**) Temperature and incubation time. (**b**) Temperature and pH. (**c**) Temperature and agitation. (**d**) Incubation time and pH. (**e**) Incubation time and agitation. (**f**) pH and agitation.

The analysis of the variance of the quadratic regression model (Table 4) indicated that the model was highly significant, as evidenced by the Fisher's F-test (F_model_ = 539.72) and a low probability value (P_model_ < 0.0001). The quadratic model also suited the data well, as evidenced by the p-value for "lack of fit" (0.2481). A, B, C, D, AC, AD, BC, BD, CD, A^2^, B^2^, C^2^, and D^2^ were the important model terms in this model. The model's R^2^ was found to be 0.9980, indicating that it was reliable. To better comprehend the interplay between independent factors, 3D response surfaces were created. Figure 3a–f depicts the response surfaces generated for the variation of cell concentration as a function of values of two variables, with the other two variables fixed to their center values. The optimum values of the respective components were represented by the coordinates of the center point within the highest contour levels in each of the figures. Response surface curves can be used to determine the range of optimum conditions within the experimental domain or to direct future experiments for better results. As shown in Fig. 3a, the temperature showed a parabolic response at distinct periods of incubation, with the maximum cell concentration attained at the temperature of 35 °C. Temperatures that were extremely low or extremely high were not conducive to cell proliferation. The variation in incubation time followed a parabolic curve as well, with optimum cell growth occurring between 32 and 36 h. Likewise, the response behavior was investigated between temperature and pH (Fig. 3b), and where the temperature had an effect on cell concentration, which followed a parabolic curve, and the optimum temperature was 35 °C. The other element, pH, had an impact on the response as well, and the maximum cell concentration was found in the range of 7 to 7.5. In Fig. 3c, the optimum temperature was around 35 °C. The agitation speed affected the cell concentration, which followed a parabolic curve. The cell concentration was lowest at very low and very high agitation rates, while the highest count was achieved between 250 and 275 rpm. As shown in Fig. 3d, the optimum cell concentration was achieved when the incubation time and pH were 32 to 36 h and 7 to 7.5, respectively. Figure 3e illustrates the response pattern of incubation periods and agitation, which was found to be parabolic, with the optimum cell concentration in the middle values. In terms of agitation, the response pattern was parabolic, as illustrated in Fig. 3f. When it came to pH, the highest cell concentration was found around the midpoint, and raising the pH had no discernible influence on the cell concentration.

**Table 4:** ANOVA for the CCD quadratic model.

Analysis of variance table [partial sum of squares—type iii]						
SOURCE	Sum of Squares	df	Mean Square	*F* value	*p* value	Significance
Model	92.23	14	6.59	539.72	< 0.0001	Significant
A-Temperature	0.5251	1	0.5251	43.02	< 0.0001	
B-Incubation time	0.2501	1	0.2501	20.49	0.0004	
C-pH	2.58	1	2.58	211.43	< 0.0001	
D-Agitation	0.1001	1	0.1001	8.20	0.0118	
AB	0.0163	1	0.0163	1.33	0.2665	
AC	0.1243	1	0.1243	10.18	0.0061	
AD	1.27	1	1.27	104.15	< 0.0001	
BC	0.2525	1	0.2525	20.69	0.0004	
BD	0.6683	1	0.6683	54.75	< 0.0001	
CD	0.2328	1	0.2328	19.07	0.0006	
A^2^	68.59	1	68.59	5619.33	< 0.0001	
B^2^	2.00	1	2.00	163.94	< 0.0001	
C^2^	6.59	1	6.59	539.76	< 0.0001	
D^2^	27.30	1	27.30	2236.36	< 0.0001	
Residual	0.1831	15	0.0122			
Lack of fit	0.1449	10	0.0145	1.90	0.2481	Not significant
Pure error	0.0382	5	0.0076			
Cor total	92.41	29				

STD. DEV. 0.1105, MEAN 7.10, C.V. % 1.56.

R^2^ 0.9980, ADJUSTED R^2^ 0.9962, PREDICTED R^2^ 0.9904, ADEQ PRECISION 84.7552.

### Verification test

The optimum values of the independent variables were predicted using the point prediction function of the design-expert software, as shown in Supplementary Table T5. The Maximum cell concentration of 9.78 × 10^8^ CFU/mL predicted was at optimal values of temperature 35 °C, incubation time 32 h, pH 7.5, and agitation speed 250 rpm. Under the predicted optimal parameter values, the maximum experimental cell concentration was 9.84 × 10^8^ CFU/mL in laboratory-scale bioreactors (FUS-10 L). The pilot bioreactor (V1: 300 L) was then operated three times under the optimized parameters, resulting in an average cell concentration of 2.05 × 10^9^ CFU/mL. Finally, using these optimized parameters, an industrial bioreactor (V2: 3000 L) was operated three times, obtaining an average cell concentration of 2.01 × 10^9^ CFU/mL, demonstrating a strong correlation in the scale-up of optimized parameters from the lab scale to the industrial scale (Supplementary Fig. F4).

### Cell concentration stability in liquid formulation

**Figure 4:**
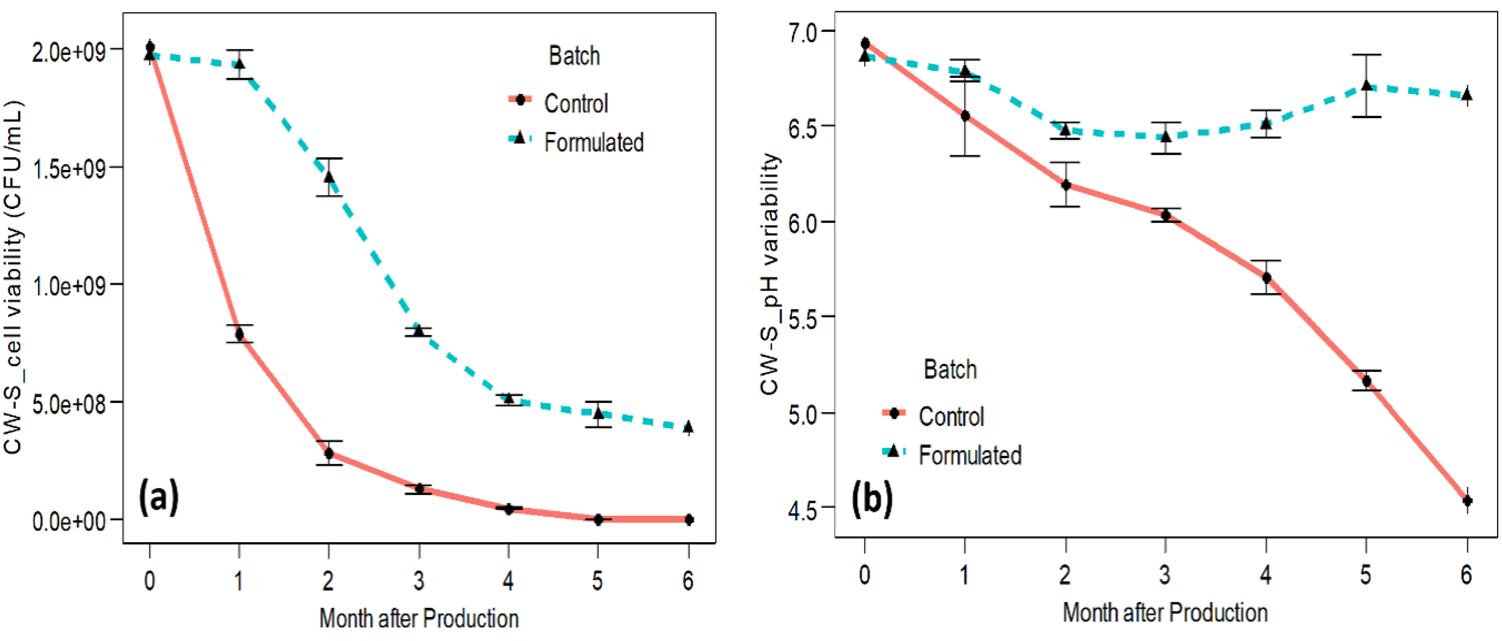
Variation in *Bacillus subtilis* CW-S cell concentration and pH in the formulation and control over time. (**a**) Viability of cell concentration (CFU/mL). (**b**) Variability in pH.

Figure 4a shows the comparative viable cell concentration of formulated and control CW-S (check culture) up to a storage period of 180 days. A significant difference in viable population was observed between formulated and control CW-S within 30 days of storage. The results showed that the formulation supported more than 3.8 × 10^8^ (CFU/mL) after 180 days of storage (Supplementary Table T6). *Bacillus subtilis* CW-S slowly died with the decrease in pH in the harvested culture of the control (Fig. 4b).

### Minituber cultivation in aeroponic

**Figure 5:**
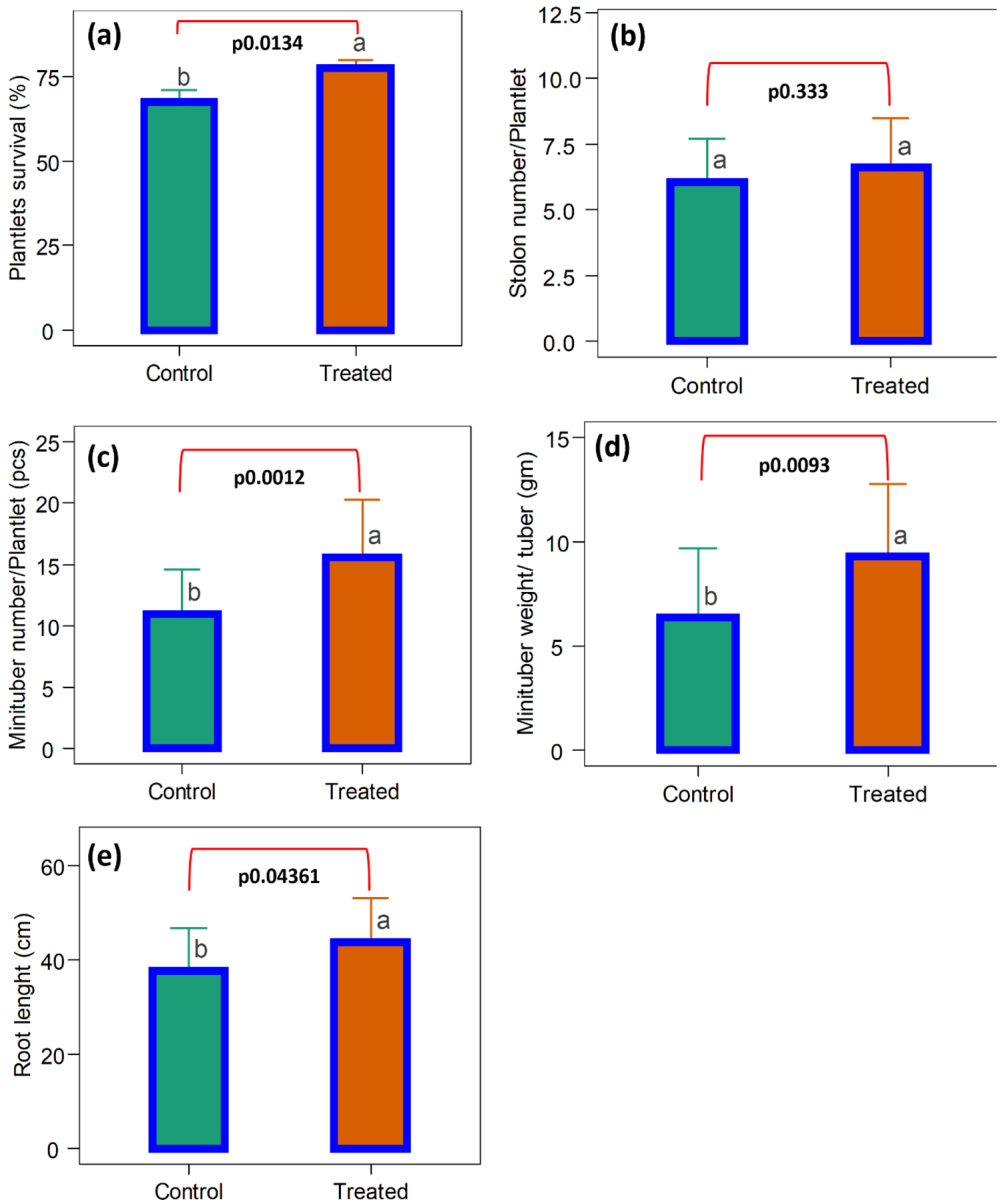
The effects of CW-S on minituber production in aeroponic. (**a**) Plantlet survival rate after transplantation in aeroponic. (**b**) Stolon number. **c**) Minituber number. (**d**) Minituber weight. (**e**) Root length. Bars without shared letters indicate significant differences between the control and treated ones.

**Figure 6:**
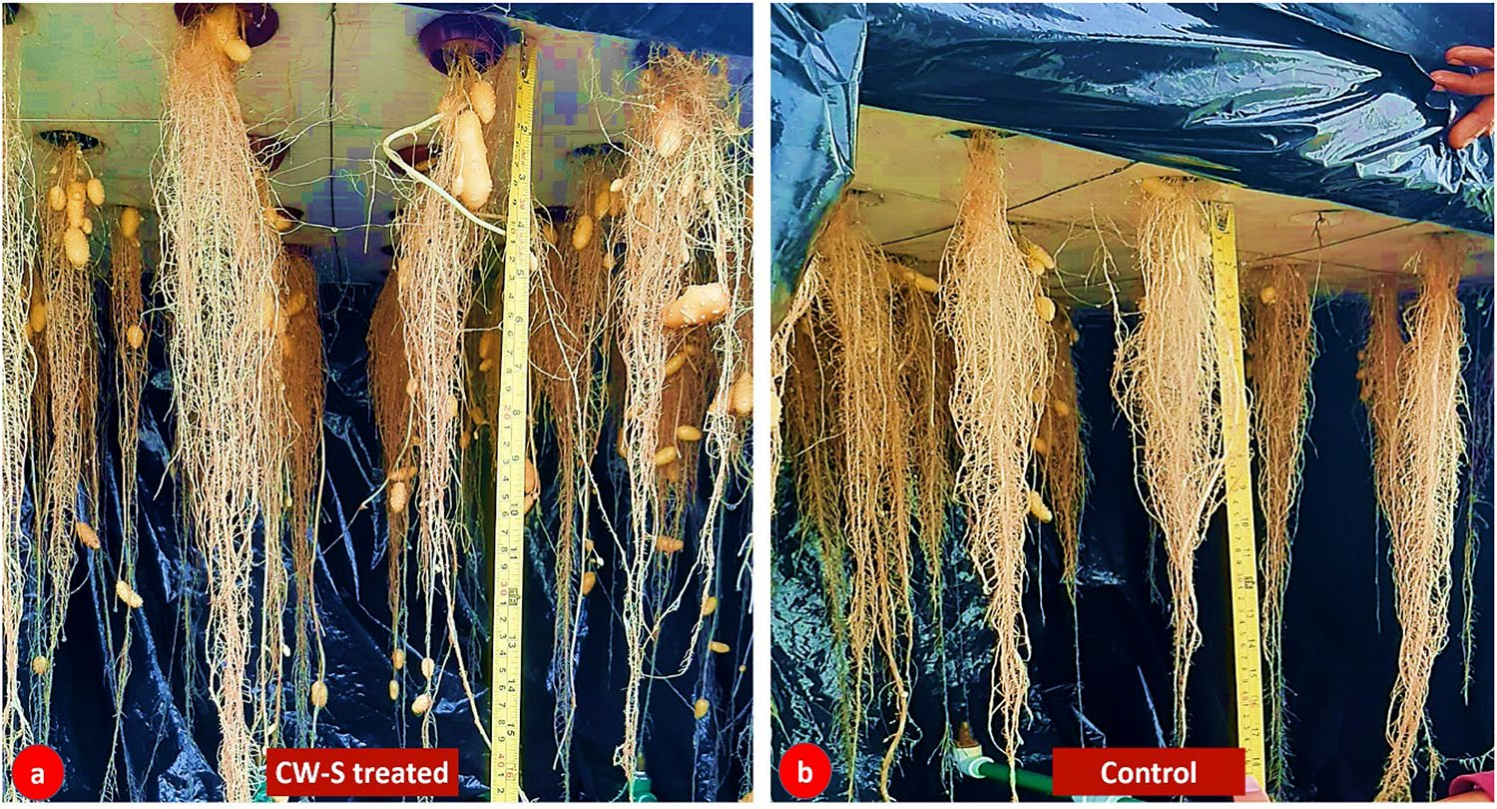
Root and stolon development inside an aeroponic system. (**a**) Potato plantlets treated with CW–S. (**b**) Potato plantlets under control.

Figure 5 illustrates the effects of CW-S on aeroponic minituber production. In treated plants, the transplantation shock was reduced, and the survival rate was much higher (77.7 ± 2.32%) than in control plants (67.5 ± 3.48%). Minituber quantity (15.6 minitubers/plant), weight (9.29 g/tuber), and root length (46.13 cm) were all increased significantly in treated plants. No significant effect was observed on the stolon number (Fig. 6). During seven days of nutrient feeding and recycling, the cell concentration varied from 5.62 × 10^3^ to 6.57 × 10^4^ CFU/mL (Supplementary Table T7). In an ELISA test, no virus was found in either the control or the treated minituber. At the end of aeroponic cultivation, 14,516 minitubers were harvested from 1,313 surviving plantlets in the control and 23,218 from 1,510 surviving plantlets in the CW-S treatment. The data shows that the overall number of minitubers in the CW-S treatment was significantly higher due to the higher survival rate of the plantlets and the increased number of minitubers per plantlet. The higher weight of the minitubers in the treated plantlets can also influence the number of minitubers by increasing the frequency of harvesting. The CW-S can be used as a plant probiotic in an aeroponic system to produce large quantities of high-quality minituber.

### Industrial-grade potato cultivation in field conditions

**Figure 7:**
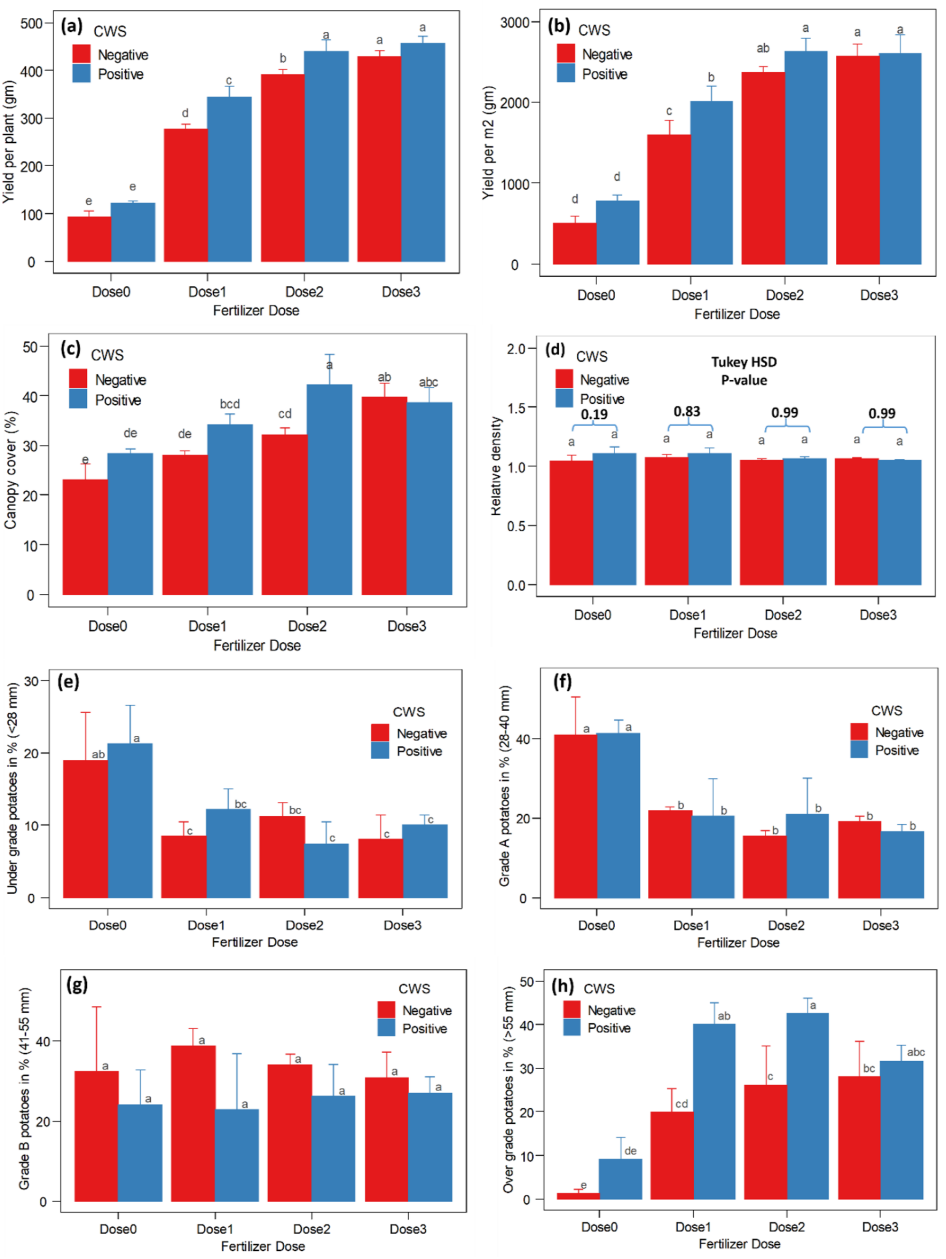
Two-way ANOVA illustrates the effect of CW-S with different fertilizer doses. (**a**) Yield per plant. (**b**) Yield per m^2^. (**c**) Canopy cover. (**d**) Relative density. (**e**) Under-grade potato percentage. (**f**) Grade A potato percentage. (**g**) Grade B potato percentage. (**h**) Over–grade (industrial-grade) potato percentage. (^ns^ *p* ≥ 0.05, * *p* < 0.05, ** *p* < 0.01, *** *p* < 0.001), data are means ± SD (*n* = 4). Bars without shared letters indicate significant differences.

**Figure 8:**
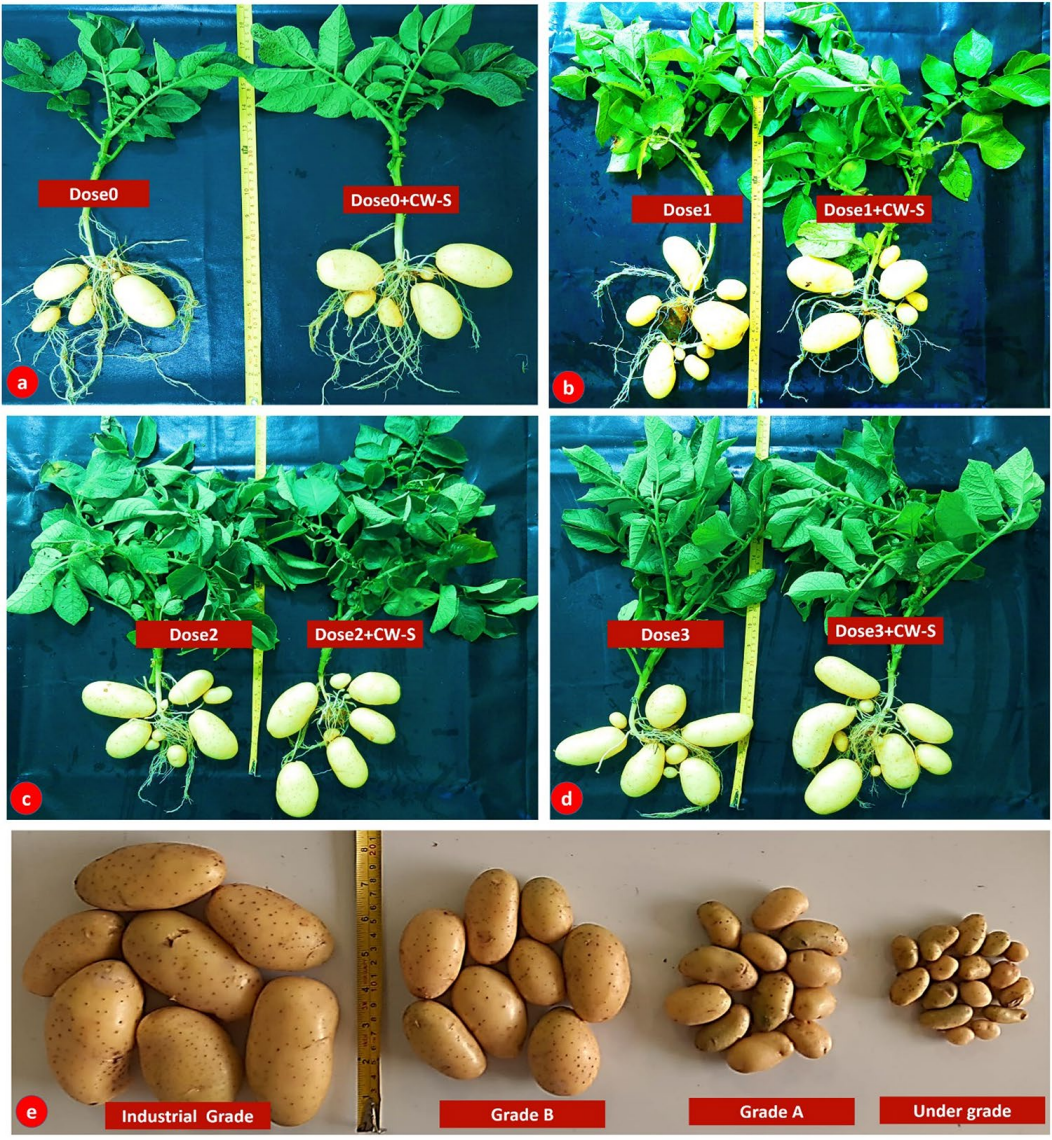
A comparison of CW-S and control in terms of yield and grade of potatoes. (**a**) Dose0 and CW-S. (**b**) Dose1 and CW-S. (**c**) Dose2 and CW-S. (**d**) Dose3 and CW-S. (**e**) A comparison of various grades.

Experiments with various NPK doses demonstrated that the CW-S enhanced yield and yield-related metrics in potato (*Solanum tuberosum*) (Fig. 7). However, yield per plant improved significantly in T6 (Dose1 + CW-S) and T7 (Dose2 + CW-S), but yield per square meter improved significantly only in T6 (Dose1 + CW-S), and canopy coverage expanded in T7 (Dose2 + CW-S). T5 (Dose0 + CW-S) demonstrated a slight increase in relative density and no adverse effects from the other treatments. In potato production, none of the CW-S treatments had a substantial impact on under-grade, grade A, or grade B. On the other hand, CW-S treatments T6 and T7 significantly enhanced the yield of industrial-grade (> 55 mm) potatoes, while other CW-S treatments showed minor improvements (Fig. 8).

In the analysis of features for industrial use, Table 5 shows that the specific density and dry matter were slightly increased, while the reducing sugar content remained unaltered, and all the treatments had no effect on the other parameters. In lab fry tests, there was also no detrimental impact. As a result, at the farmer level, we can combine CW-S with dose1 or dose2 to reduce the use of chemical fertilizer while increasing the productivity of industrial quality (> 55 mm) potatoes for making French fries, chips, and other products.

**Table 5:** An assessment of the industrial parameters of potatoes grown with CW-S.

Parameter	Standard Specification	UoM	T1 (Dose0)	T2 (Dose1)	T3 (Dose2)	T4 (Dose3)	T5 (Dose0 + CW-S)	T6 (Dose1 + CW-S)	T7 (Dose2 + CW-S)	T8 (Dose3 + CW-S)
Eyes	Shallow	N/A	Deep	Deep	Deep	Deep	Deep	Deep	Deep	Deep
Tuber shape	Round to oval	N/A	Oval (Long Shaped)	Oval (Long Shaped)	Oval (Long Shaped)	Oval (Long Shaped)	Oval (Long Shaped)	Oval (Long Shaped)	Oval (Long Shaped)	Oval (Long Shaped)
Sp. Gravity %	≥ 1.08	%	1.042 ± 0.049	1.072 ± 0.024	1.052 ± 0.014	1.063 ± 0.009	1.1062 ± 0.06	1.107 ± 0.051	1.065 ± 0.015	1.051 ± 0.006
Dry matter content	≥ 20	%	19.248 ± 0.154	19.968 ± 0.136	19.796 ± 0.099	20.114 ± 0.156	20.31 ± 0.426	20.720 ± 0.343	20.706 ± 0.410	20.758 ± 0.389
Reducing sugar (Glucose)	≤ 15 mg/dL	mg/dl	12.576 ± 0.157	12.634 ± 0.160	12.728 ± 0.221	12.638 ± 0.197	12.586 ± 0.159	12.738 ± 0.194	12.72 ± 0.225	12.756 ± 0.205
Texture	Fairly firm to mealy	N/A	Ok	Ok	Ok	Ok	Ok	Ok	Ok	Ok
Presence of Vascular ring	The vascular ring should not appear dark with diseases	N/A	Ok	Ok	Ok	Ok	Ok	Ok	Ok	Ok
Flesh Color	Pale/ Creamish	N/A	Creamish	Creamish	Creamish	Creamish	Creamish	Creamish	Creamish	Creamish
Defects	Skin/Peel should not be feathering type when supplied from Cold Store	N/A	Ok	Ok	Ok	Ok	Ok	Ok	Ok	Ok
Maturity	Fully matured	N/A	Mature	Mature	Mature	Mature	Mature	Mature	Mature	Mature
Lab fry test	Creamish to light Brown	N/A	Creamish	Creamish	Creamish	Creamish	Creamish	Creamish	Creamish	Creamish
Appearance	Should be free from any type of damage & Sprouts	N/A	Ok	Ok	Ok	Ok	Ok	Ok	Ok	Ok

Tuber Temperature = 27.40 °C = 81.32 °F. Defect = 00%

Test Report (In loading Point): R/S = x mg/dl, DMC = x%; Temp: x °F = x °C; DT = 0:0 pm; RT = 02:00 pm.

## Discussion

Several challenges must be overcome to develop an effective commercial plant probiotic product, including isolation, screening, production, formulation, and field evaluation. Active agent manufacturing must use low-cost raw materials to be both cost-effective and high-quality and demonstrate good field performance^27^.

During the isolation and screening process, the active agent should have several plant growth-promoting characteristics. In this research, *Bacillus subtilis* CW-S was isolated from a wheat crop field, indicating that it could be a plant-beneficial microbe^28^. Plant probiotic assays revealed that the strain CW-S was capable of producing IAA, solubilizing phosphate, fixing nitrogen, and demonstrating antagonism against phytopathogens such as *Curvularia spicifera*, *Alternaria alternate*, *Sclerotium theobromae*, and *Fusarium equiseti*. In an in-vitro seedling assay, this strain promoted plant growth by significantly increasing the vegetative growth parameters of wheat seedlings. The plant probiotic activity of this strain was comparable to that described in past studies, which showed that *Bacillus subtilis* is a rhizospheric bacteria with numerous favorable effects on crops and plants^2,29,30^.

Since microbes have a wide range of physiological characteristics, their growth is highly influenced by media ingredients, particularly carbon and nitrogen sources^31^. Molasses was chosen as a favorable substrate as it has been used to grow several microbes, including *B. subtilis*^32–34^. Due to its high sugar content, colloidal suspension, vitamins, and nitrogen compounds, molasses is often used in the production of microorganisms^26^. Urea was selected as a nitrogen source since it has been utilized in the cultivation of microorganisms by several authors^35,36^. In our media evaluation experiments, molasses and urea were found to be suitable sources of carbon and nitrogen, respectively. We were able to improve cell concentration (2.01 × 10^9^ CFU/mL) by using simple carbon and nitrogen sources, and the results shown here are comparable to those previously reported by Yánez-Mendizábal et al. (2012; 3 × 10^9^ CFU/mL) and Khardziani et al. (2017; 2.3 × 10^9^ CFU/mL)^34,37^.

Additional parameters such as pH, temperature, agitation, and aeration, in combination with carbon and nitrogen inputs, play an important role in the growth of *B. subtilis*^34,38^. Response surface methodology is a powerful statistical tool for analyzing and evaluating the interactions of various process factors^39^. In the current study, statistical process optimization using PBD and CCD resulted in a significantly higher cell concentration (2.01 × 10^9^ CFU/mL), which was found to be comparable to prior research^39–42^. Large-scale manufacture of CW-S is necessary for commercial application at a lower cost. Several studies explored the production process in various volumes ranging from 1 to 70L to obtain large amounts of biomass and secondary metabolites^39,42–44^. In this study, the CW-S manufacturing process was evaluated on a lab (10 L), pilot (300 L), and industrial (3000 L) scale. The evaluation results revealed that the optimized process performed well at an industrial scale (2.01 × 10^9^ CFU/mL), with increased cell concentrations than in the laboratory (9.84 × 10^8^ CFU/mL) bioreactors. This will be the first report of *Bacillus subtilis* production process evaluation on an industrial scale of 3000 L. This could pave the way for large-scale commercial production of *Bacillus subtilis* at a lower cost.

In the development of microbial products, shelf life and microbe viability are critical considerations. In our experiments, polymeric materials including polyvinylpyrrolidone (PVP), carboxymethyl cellulose (CMC), and the surfactant Polysorbate 20 added to the formulation increased cell viability. The formulation supported > 3.8 × 10^8^ CFU/mL of CW-S for more than 180 days. PVP, a nontoxic polymer, might provide a favorable environment for bacterial survival, and CMC functions as an adjuvant^66^.

According to several reports, aeroponic systems are becoming a popular technology for producing high-quality minituber^45,46^. In this study, minitubers were produced in an aeroponics system in which CW-S was applied with the nutrient spray and foliar spray. The minituber number, weight, and root length all increased significantly in treated plants when compared to controls. The number of tubers produced by treated plants was 15.6 per plant, which was reasonable and within the range reported by Farran et al. (2006) of 13.4 per plant^47^. Our results in terms of tuber number and average tuber weight were not comparable to those reported by others since we used a different cultivar^48^. The majority of the researchers cited did not mention root length, stolon number, or transplanted plantlet survival rates, and there was no evidence of the use of plant probiotics in aeroponics such as *Bacillus subtilis*^46–49^. *Bacillus subtilis* CW-S performed well in aeroponic because it offered plants biotic and abiotic stress tolerance via induced systemic resistance (ISR), biofilm growth, and lipopeptide production^30,50^.

In field conditions, CW-S improved potato yield and yield-related parameters (yield per plant, yield per m^2^, canopy coverage, and industrial-grade potato) while using less fertilizer than the control (untreated). The majority of *Bacillus subtilis* experiments were focused on disease biocontrol^49,51^. *Bacillus subtilis* has not been evaluated in terms of the production of various potato grades utilized in the industry. According to this study, CW-S was able to increase industrial-grade potato yield without altering its industrial properties.

The findings of this study revealed that manufacturing is an important phase in the development of a plant probiotic product, with low-cost medium design, optimization, and scaling-up of the production process all contributing to the design of large-scale production. The efficacy of the product in novel applications such as aeroponic minituber and industrial-grade potato production is a good indication of its economic viability. From this perspective, the current study provides evidence for *B. subtilis* CW-S industrial production to deliver a competent plant probiotic product.

## Materials and methods

### Isolation of *Bacillus subtilis* (Strain: CW-S)

The plant probiotic *B. subtilis,* strain CW-S, was isolated from the rhizospheric soil of a wheat field in Nilphamari, Bangladesh (26°15′56.1"N 88°54′22.4"E). A diluted rhizospheric soil sample was treated with heat (80 °C for 10 min) and 1% NaAz (sodium azide). Heat-treated soil suspensions were incubated for 2 h at room temperature. Then it was serially diluted before being spread on LB agar for single colony isolation following the Tran et al. (2021) spreading method. Single colonies were recovered after 26 h of incubation at 28 ± 2 °C^4^. To assure spore-producing ability, single colonies were plated on sporulation media. *Bacillus sp.* produces oval endospores that can be dormant for long periods in stressful environments. A microscopic examination of endospores and spores, as well as rod-shaped cells, under a phase-contrast microscope (Axio Imager A1, Carl Zeiss, Germany) at 640X was carried out. The isolates were gram-stained, and the gram-positive ones were picked for the MR-VP test*.* After that, the isolates were tested for starch hydrolysis, catalase, and citrate activity, as well as growth in 7.0% NaCl-containing NA media at 45–50 °C. Those that showed positive results in this medium were selected. By streaking the isolates on *Bacillus* differentiation agar media containing Bromocresol purple, they were additionally evaluated for Mannitol fermentation ability. In this media, Bromocresol purple was utilized as a pH indicator to detect mannitol fermentation. To obtain pure cultures, single colonies were picked and re-streaked on *Bacillus* differentiation agar media (HI-Media Laboratories Private Limited, LBS Marg, Mumbai, India). Before using sequencing technology to identify bacteria, these biochemical tests were necessary for primary identification^4,52^. All methods were performed in accordance with the relevant guidelines and regulations.

### Molecular characterization (confirmatory test) of *Bacillus subtilis* CW-S

A single colony of CW-S was utilized to extract genomic DNA using the PrepManUltra® reagent (Applied Biosystems), and DNA concentration was measured using a spectrophotometer. A working solution of 1 ng/μl was made by diluting a stock solution of DNA. On the MyGeneTM Series Peltier Thermal Cycler (LongGene Scientific Instruments Co., Ltd., Hangzhou, China), the three fragments of the 16S ribosomal RNA gene were amplified using the MicroSeq® Full Gene 16S rDNA Bacterial Identification PCR Kit. Before sequencing, the amplified products were purified using ExoSAP-IT® reagent (USB) according to the manufacturer's instructions. Cycle sequencing for each amplified product was performed using the MicroSeq® Full Gene 16S rDNA Bacterial Identification Sequencing Kit. The cycle sequencing products were purified using ethanol precipitation before being delivered to the National Institute of Biotechnology (NIB) in Savar, Dhaka, where they were dissolved in HiDi formamide (Applied Biosystems) and examined on a 3130 Genetic Analyzer (Applied Biosystems). The sequence was documented in detail in the NIB report. The evolutionary history was estimated using the Maximum Likelihood approach and the Tamura-Nei model^53^. It displayed the tree with the greatest log likelihood (−13,678.84). The initial tree(s) for the heuristic search were built automatically using the Neighbor-Join and BioNJ algorithms on a matrix of pairwise distances estimated using the Tamura-Nei model, and then selected the topology with the highest log-likelihood value. In this investigation, ten nucleotide sequences were employed, with the first + second + third + noncoding codon positions included. In total, 1456 positions were found in the final dataset. MEGA X was used to conduct an evolutionary analysis^54^.

### Assay of *Bacillus subtilis* CW-S for plant probiotic potential

- **Qualitative measurement of nitrogen-fixing ability (In-Vitro)**
  To test nitrogen-fixing ability, the culture was streaked over a semi-solid nitrogen-free malate (Nfb) medium plate and incubated at 30 ± 2 °C for 72 h^55^. By exploiting free nitrogen, bacteria with nitrogen-fixing capacity might grow in this medium.
- **Quantitative estimation of phosphate solubilization (available P)**
  The Vanadomolybdophosphoric Yellow color method in the nitric acid system was slightly modified for the quantitative estimation of solubilized P by CW–S^56^. In a 100 mL volumetric flask, 0.02195 g of dipotassium hydrogen phosphate (K_2_HPO_4_) was dissolved in 40 mL of distilled water. Then 2.5 mL of 7 N H_2_SO_4_ was added, mixed, and diluted with distilled deionized water to the desired concentration, yielding 50 ppm of P. This was equivalent to a concentration of 50 mg/L (0.05 mg/mL). In each test tube, 2 mL of a mixture of solutions A (25 g/L ammonium molybdate [(NH_4_)_6_Mo_7_O_24_.4H_2_O]) and B (1.25 g/L of ammonium metavanadate [NH_4_VO_3_] and 250 mL of HNO_3_) were added to a phosphate stock solution with the following concentrations: (0.00, 6.25, 12.5, 25.0, and 50.0 ppm). After 10 min, the intensity of the yellow color formed was spectrophotometrically measured at 490 nm (BK-D590 Double Beam Scanning UV/VIS Spectrophotometer). The standard curve was created by plotting absorbance at 490 nm against P concentration. Single CW-S colonies were used to inoculate LB broth in conical flasks, which were then incubated for 48 h at 30 °C and 180 rpm. For the next step, Pikovskaya's medium (PKV Broth) was prepared, as well as a control (sample blank) that was not inoculated with bacterial culture. Using the spread plate technique, the CFU of CW-S produced in LB media were counted. In 250 mL conical flasks, an equivalent quantity of cells (10^8^ CFU) was inoculated into 50 mL of PKV broth. After that, the flasks were incubated at 30 °C for 48 h at 180 rpm. At the same time, PKV was incubated as a medium control under the same conditions. After 72 h, 1 mL of bacterial cultures were centrifuged for 10 min at 10,000 rpm from each inoculated and control PKV broth. After centrifugation, the supernatants from the control and inoculated PKV cultures were diluted ten times with 200 µL of supernatant and 1800 µL of distilled water. After that, 2 mL of final reagent (Solution A + Solution B) was added and incubated at room temperature for at least 20 min. Finally, using a spectrophotometer to measure optical density (OD) at 490 nm, the amount of P-solubilized was estimated from the standard curve.
- **Quantitative estimation of Indole 3 acetic acid (IAA) production**
  With slight modifications, Indole-3 Acetic Acid was determined using Bric et al. (1991)^57^.To determine IAA production, isolates were inoculated in Nutrient broth and incubated at 29 ± 2 °C for 48 h. 1 mL of inoculated culture was placed in 10 mL of fresh broth containing 2 mg/mL of L-Tryptophan and incubated for 72 h at 29 ± 2 °C. Approximately 2 mL of culture solution was centrifuged at 15,000 rpm for 1 min and the supernatant was used to detect IAA concentration. One mL of the supernatant was mixed with 2 mL of Salkowski’s reagent according to Gordon and Weber (1951)^58^. After 25–30 min, the absorbance of each solution was measured using a spectrophotometer at 530 nm (BK-D590 Double Beam Scanning UV/VIS Spectrophotometer). Making a series of pure IAA solutions (0, 5, 10, 15, 20, 25, 30, 35, 40, 45, 50, 55, 60, and 65 g/mL of pure IAA in each solution) and measuring the absorbance of these solutions in a spectrophotometer at 530 nm generated an IAA standard curve. The concentration of IAA produced by CW-S was determined by plotting it on this standard graph. As a control, supernatants from uninoculated test tubes were employed, and no apparent color was noticed.

### Antagonistic activity of CW-S (*Bacillus subtilis*)

3 $$ {\text{I }} = \, ({\text{D}}_{0} - {\text{D}}_{{\text{b}}} )/{\text{D}}_{0} \, \times \,{1}00 $$

CW-S was tested against phytopathogens such as *Curvularia spicifera* (NCBI Accession No: MK478831), *Lasiodiplodia theobromae* (MK478824), *Sclerotium delphinii* (MK478832), *Fusarium equiseti* (MK478826), and *Alternaria alternata* (MK478825) (available at Apex Biotechnology Laboratory culture collection) using a dual culture approach^59^. A 5 mm diameter plug containing mycelium from five-day-old targeted phytopathogenic fungi was placed in the center of the PDA plates. Single CW-S bacterial colonies were patched around 3 cm away from the fungus. The plate was incubated in the dark for seven days at 30 ± 2 °C after inoculation. At least ten replications were used in the experiment. CW-S antagonistic activity was expressed as an inhibition percentage (I). It was calculated using Eq. (3).

Where D_0_ represents the growth diameter (cm) of the fungus on the control side and D_b_ denotes the diameter (cm) of the fungus when challenged with CW-S (Supplementary Fig. F5).

### PGPR- seedling assay: for plant growth promoter rhizobacteria (PGPR)

After washing with sterile distilled water (1–2 times), the wheat seeds were surface sterilized by dipping in 3% sodium hypochlorite (5 min), followed by dipping in 95% ethanol (20 s; from dipping to washing with distilled water), and finally washing 7 times with sterile distilled water. The soil was disinfected after 1 h of autoclaving at 121 °C and 15 psi. The autoclaved soil was oven-dried for 48 h at a constant temperature (70 ± 2 °C) before being filled into experimental pots. There was 200 g of dried soil in each container. In each pot, three plants were sown. There were two treatments: T_1_ for treated seedlings (CW-S) and T_0_ for non-inoculated seedlings (control). For each treatment, 20 randomized replication pots were used in this experiment. All plants were grown in a plant growth chamber (BIOBASE: BJPXA450) at 25 °C and 68 ± 2% relative humidity. Extra plants were removed from the pots at 6 DAS (Days after sowing) while keeping one plant in each pot. After sowing seed in the pot, 2 mL of CW-S was fed through a micropipette for inoculation. Plant development was aided by the application of 2 ml of half-strength Hoagland feeding solution without phosphorus (P) to each pot at three-day intervals^60^. After 35 DAS, ten competitive seedlings were harvested from each treatment for data collection.

### Culture media for growth in submerged bioreactors

For lab-scale submerged culture, LB media (Luria Bertani broth) was utilized, but for large-scale production, a novel medium was required. The goal was to develop a low-cost media that could yield higher cell concentrations, maintain cell concentration and pH throughout storage, and perform well in plant assays, with locally available materials like molasses (Rajshahi Sugar Mill Ltd., Harian, Rajshahi, Bangladesh) and urea being used (Karnafuli Fertiliser Company, Chittagong, Bangladesh). Biofertilizer production demands a high biological output (CFU/unit). As a control, the LB broth medium was used, which contains expensive nitrogen sources. The goal of the study was to see how different carbon and nitrogen sources affected CFU output, as well as to select low-cost carbon and nitrogen sources. CW-S (*Bacillus subtilis*) was first cultured on 10 different specifically developed production media [BS1: Sucrose (10 g/L), (NH_4_)_2_HPO_4_ (1 g/L); BS2: Dextrose (10 g/L), (NH_4_)_2_HPO_4_ (1 g/L); BS3: Glycerol (10 g/L), (NH_4_)_2_HPO_4_ (1 g/L); BS4: Starch (10 g/L), (NH_4_)_2_HPO_4_ (1 g/L); BS5: Molasses (20 g/L), (NH_4_)_2_HPO_4_ (1 g/L); BS6: Sucrose (10 g/L), CO(NH_2_)_2_ (2 g/L); BS7: Dextrose (10 g/L), CO(NH_2_)_2_ (2 g/L); BS8: Glycerol (10 g/L), CO(NH_2_)_2_ (2 g/L); BS9: Starch (10 g/L), CO(NH_2_)_2_ (2 g/L); BS10: Molasses (20 g/L), (CO(NH_2_)_2_ (2 g/L)] , in parallel to LB medium, to comprehend how they affected biological yield. Media BS1 to BS10 were supplemented with MgSO_4_.7H_2_O (0.03 g/L); Defatted soybean meal (1.25 g/L); Soybean oil (2 mL/L) as an antifoaming agent and the proportions of the buffering components [K_2_HPO_4_ (0.025 g/L); KH_2_PO_4_ (0.025 g/L)]. The media evaluation was carried out in a 250 mL Erlenmeyer flask containing 50 mL of each medium with three replications. The flasks were inoculated with 1 mL of freshly prepared inoculum and kept at 30 ± 2 °C under constant shaking at 180 rpm in a shaking incubator. All flasks were removed at 36 h and analyzed for colony-forming unit (CFU) tests.

### Estimation of the total carbohydrate content of molasses samples

Total carbohydrate content was determined using the colorimetrical anthrone method^61^. Molasses samples were treated with 2% anthrone solution in 98% H_2_SO_4_. The samples were then heated for 10 min in a boiling water bath. The absorbance of the samples was measured spectrophotometrically at 578 nm using the Tomos Biotools (Shanghai) Co., Ltd., UV-3300PC after cooling them to room temperature. The amount of carbohydrate (mg/mL) was calculated using the standard calibration curve for glucose. The carbohydrate content was measured in percentage (m/m).

### Seed culture preparation

Single colonies were recovered from a glycerol-preserved culture after 20 h of incubation at 35 °C. The initial broth culture was produced from single colonies in a flask of 250 mL containing 50 mL of the medium after 20 h of incubation at 35 °C and 200 rpm. After that, the broth culture was subcultured in the same conditions. Finally, seed culture was obtained from that subculture after 20 h of incubation at 35 °C and 200 rpm in a flask of 1000 mL containing 300 mL of LB media.

### Selection of significant independent factors affecting the cell concentration of *B. subtilis* CW-S by PBD

4 $$ {\text{y }} = {\text{ a}}_{0} + {\text{ a}}_{{1}} {\text{x}}_{{1}} + {\text{ a}}_{{2}} {\text{x}}_{{2}} + \cdots + {\text{ a}}_{{{11}}} {\text{x}}_{{{11}}} $$

A PBD is a filtering design that is effective in identifying critical factors among a vast number of factors that ultimately affect the outcomes. With a total of 12 experimental trials created by the software, the PBD was used to identify important factors that influence *Bacillus subtilis* (CW-S strain) cell concentration (Table 2). A first-order quadratic model was used to filter a large number of independent factors and pick a smaller number for further optimization, allowing us to study n-1 factors with only n experiments. The difference between the average response value measured at a high (+ 1) and low (−1) setting was used to calculate the major effect for each factor. For further optimization, the CCD statistical model was used to take into account the factors that had a significant impact on cell concentration. Agitation speed (A), aeration (B), inoculum size (C), carbon source percentage (molasses; D), nitrogen source percentage (urea; E), the incubation period (F), pH (G), and temperature (H) were all taken into account, with their high and low ranges displayed in Table 6. The upper and lower boundaries of each variable's range were defined by these levels. Equation (4) shows the PBD based on a first-order polynomial model for mathematical modeling that was used:

**Table 6:** High and low levels of factors used in PBD.

S/N	Coded factors	Factors	Low level (−1)	High level (+ 1)
1	A	Agitation speed (rpm)	150	300
2	B	Aeration (L/h)	8	10
3	C	Inoculation Percentage (v/v ;%)	2	3
4	D	Carbon source percentage (Molasses; (w/v ;%)	2	3
5	E	Nitrogen source percentage (Urea; w/v ;%)	1.5	3
6	F	Incubation time (hours)	24	36
7	G	pH	6	8
8	H	Temperature (°C)	28	40

Where ‘‘y’’ represents the predicted response, x_1_–x_11_ represent the parameters (factors), a_1_–a_11_ are the relevant coefficients, and a_0_ represents the intercept of the mean. The significant independent variables with a probability value (p value < 0.05), that is, greater than 95% of the confidence level of all the intervals, are deemed to have a substantial effect on growth^62,63^.

### Optimization of significant parameters using RSM

5 $$ {\text{Y }} = \, \upbeta_{{\text{o}}} + \, \upbeta_{{1}} {\text{X}}_{{1}} + \, \upbeta_{{2}} {\text{X}}_{{2}} + \, \upbeta_{{{12}}} {\text{X}}_{{1}} {\text{X}}_{{2}} + \, \upbeta_{{{11}}} {\text{X}}_{{1}}^{{2}} + \, \upbeta_{{{22}}} {\text{X}}_{{2}}^{{2}} + \,\varepsilon{} $$

The PBD's significant variables were fed into the CCD Software Design Expert, a popular quadratic test design for sequential test development that uses regression analysis to forecast component levels for optimal response. Temperature, pH, incubation period, and agitation were investigated at five different levels (−2, −1, 0, 1, 2) to see how they affected the cell concentration of the strain CW-S (Table 7). A total of 30 experiments were built using a full factorial central composite design with 16 (2^4^) cube points, 6 center points, and 8 (4 × 2) star points. The relationship between cell concentration and independent factors was characterized using a quadratic model based on the second-order polynomial Eq. (5)^64^.

**Table 7:** Actual and coded values of the factors in employment in the CCD.

Factors	Range of Levels					
	Code	− 2	− 1	0	1	2
Temperature (°C)	A	25	30	35	40	45
Incubation Time (hours)	B	24	28	32	36	40
pH	C	6.5	7	7.5	8	8.5
Agitation (rpm)	D	150	200	250	300	350

Where Y is the measured response (cell concentration), X_1_ and X_2_ are significant independent variables, β_12_ is the interactive regression coefficient, and β_0_ is a constant term, and β_1_ and β_2_ are the linear regression coefficients, respectively. An ANOVA was used to determine the model's significance and regression coefficient. The coefficient (R^2^) was calculated to measure the model's fit, and Fisher's F-test was performed to demonstrate statistical significance. The mutual corrections between the relevant parameters were evaluated using the response surface and contour structures of the model-expected responses^65^.

### Model validation

To validate the RSM-generated mathematical model, a three–replication experiment was conducted using the expected optimum parameter conditions. The optimized parameters were used to run the pilot and industrial bioreactor as a part of the scale-up.

### Formulation preparation and stability checking

Following the completion of the culture hour, the culture was supplemented with 2 ± 0.1% Polyvinylpyrrolidone (PVP), 0.1 ± 0.02% Carboxymethyl Cellulose (CMC), and 0.025 ± 0.005 mL/L Polysorbate 20. The culture was placed in HDPE bottles after two hours of mixing and placed under quality control to check the stability of the formulation. A check culture was also prepared with the same procedure without adding the supplements. At monthly intervals up to 180 days, the survival of *Bacillus subtilis* CW-S in a liquid formulation stored at 25 ± 2 °C in a biochemical incubator under dark conditions was studied using plate-count procedures^66^.

### Minituber cultivation (Aeroponic)

The experiment of minituber cultivation was carried out in the net-house aeroponic system at ABBL (Apex Bio-fertilizer & Bio-pesticides Ltd., Gobindaganj, Gaibandha, Bangladesh: 25°148ʹN, 89°89ʹ) (Supplementary Fig. F6). The tissue culture plantlets were made from the SANTANA cultivar (Nederlands Potato Consultative Foundation: GBR165). The experiment was divided into two blocks, one for control and the other for treatment, with each block having its own nutrient source. Each block with three racks has a total capacity of 1944 (3 × 648) plantlets. Plantlets were hardened in a pluck tray with coco-peat mixed with CW-S culture at 25 ± 2 °C. After 15 days of hardening, the plantlets were transplanted to the aeroponic racks. Then the survival rate was recorded. Every seventh day, the nutrient solutions were replaced with a new solution mixed with CW-S (5%). The concentration of CW-S cells in the nutrient solution was measured on the first and seventh days. Every 15 days, CW-S was sprayed on the leaves. After 25 days of transplantation, minitubers were harvested seven days apart. All of the minitubers were harvested after 80 days, and the data was recorded (Supplementary Fig. F7). The minituber from both the control and treatment groups was then tested for virus detection in BARI (Bangladesh Agricultural Research Institute, Joydebpur, Dhaka, Bangladesh).

- **Nutrient Solutions and Cultivation Conditions**
  In this experiment, an optimized nutrient solution was used (gL^−100^: CaNO_3_ 25, KNO_3_ 50, KH_2_PO_4_ 15, MgSO_4_ 20, Fe EDTA 0.30, and Fetrilon combi 1.5). For the first week following plantlet transplantation, half strength was utilized, but after that, full strength was used. The nutrient solution's pH and EC were 6.25–6.80 and 1.5–2.0 ms/cm, respectively. Temperatures fluctuated between 7 and 11 °C at night and 15–25 °C during the day.

### Industrial-grade potato cultivation in field conditions

The field experiment was carried out in the ABBL research field to evaluate the effect of the formulated CW-S on the yield and yield-related parameters of potato (Variety: SANTANA). Each experiment was set up in a split-plot design with four replicates. Each plot measured 6 m × 3.5 m. The study included T1-T4 (no CW-S application) and T5-T8 (CW-S application) treatments, as well as four NPK doses: no NPK (Dose0), 50% NPK (Dose1; 130, 125, and 187.5 kg/h), 100% NK (Dose2; 260, 375 kg/h), with 50% P (Dose2; 125 kg/h), and 100 percent NPK (Dose3; 260, 250, and 375 kg/h). During final land preparation, gypsum, zinc sulfate, and boron were applied as a basal application at rates of 8, 10, and 10 kg/ha, respectively. In mid-November, seeds were sown (Supplementary Fig. F8) in the field. Each plot had a seed-showing line distance of 60 cm and a plant-to-plant distance of 25 cm. Except for urea, all fertilizers were applied as a base dose during final land preparation. At three intervals, different nitrogen dosages (urea) were applied. At harvest, yield-contributing parameters and yield were recorded to determine the reduced fertilizer dose for CW-S. According to Karim et al. (2010), potatoes were divided into four grades based on the diameter of the potato in the center, including under-grade (less than 28 mm), Grade A (28–40 mm), Grade B (41–55 mm), and over-grade (industrial-grade ˃ 55 mm)^67^. The specific gravity was measured, as per Schippers (1976)^68^. Samples from each treatment (T1-T8) were evaluated according to Hassanpanah et al. (2011) and Abong et al. (2010) to investigate the impact of CW-S on the industrial features of potato^69,70^.

### Analytical techniques and quality control

Samples were taken and examined under a microscope to check for growth and contamination. The samples were subsequently streaked on NA (HIMEDIA) plates to confirm purity. After that, the plates were incubated at 35 °C for 24–48 h. If any colonies other than the typical *Bacillus* type were detected, the sample was considered to be contaminated. The cell suspensions were then diluted with dilution factors ranging from 10^–1^ to 10^–7^. After that, the diluted cell suspensions were spread on DM agar plates and incubated at 35 °C. After 48 h of incubation, the cell concentration was calculated. The spread plate technique was used to determine the cell concentration of *B. subtilis*^71^.

### Bioreactors

Three different types of bioreactors were used in this investigation, spanning from laboratory to industrial scale. For the PBD and RSM experiments, three laboratory bioreactors (FUS-10 L) were used. The optimized parameter was tested on an industrial scale with a pilot (V1:300 L) and an industrial bioreactor (V2:3000 L) made by Wenzhou Daysly Technology Co. Ltd., Zhejiang, China. The experiment was carried out at ABBL's fermentation plant (Supplementary Fig. F9), which has the facility of all utility setups, viz., an oil-free compressor (Atlas Copco, Model: ZT37, 8.6–50 HZ, capacity: 5.5 m3/min), an industrial chiller (KL-LSF30S, capacity: 25 T), an oil-generated boiler (ST 1300EF), etc. A computer-aided data bioprocessing system program (MCGS) was used to automate the process. Integrated biosensors were used to control temperature, pH, and DO (dissolved oxygen)^72,73^.

### Statistical tools

Minitab software was used to develop PBD for independent factors screening and Design Expert for designing a CCD for independent factors optimization^74,75^. A Tukey's HSD test was conducted to examine significance variation at the *p* < 0.05 level using ANOVA in R^76^.

## Supplementary Information

Supplementary Information.

## Data Availability

The datasets generated during and/or analysed during the current study are available from the corresponding author on reasonable request.
