# Supplementary material for: Optimization of industrial (3000 L) production of Bacillus subtilis CW-S and its novel application for minituber and industrial-grade potato cultivation
Source: Sci Rep. 2022 Jul 1;12:11153. doi: 10.1038/s41598-022-15366-5 (PMC9249890; doi:10.1038/s41598-022-15366-5)
Supplement: Supplementary file 1 — Supplementary Information. [file 41598_2022_15366_MOESM1_ESM.pdf]

Optimization of industrial (3000L) production of *Bacillus subtilis* CW-S and its novel application for minituber and industrial-grade potato cultivation

Md. Abuhena[1†], Jubair Al-Rashid[1,2†], Md. Faisal Azim*[1†], Md. Niuz Morshed Khan[2], Md. Golam Kabir[1,2], Nirmal Chandra Barman[2], Noorain Munim Rasul[1,2], Shahina Akter*[3] and Md. Amdadul Huq*[4]

Supplementary dataset

**Biochemical Test**	**Result**	**Remarks**
Shape	Rod	Microscopic Observation
Gram staining	+	Microscopic Observation
Starch hydrolysis	+	To test for amylase producing capabilities
Spore formation	+	Microscopic Observation
Catalase	+	key biochemical test for bacillus group
VP	+	ok
MR	-	ok
Citrate	+	ok
Indole	+	ok
7 % NaCl growth	+	Growth observed
Growth in Nitrogen free media	+	Growth observed
Growth at 45-50°C temperature	+	Growth observed
Bacillus differentiation agar	+	To test for mannitol fermentation abilities. *Bacillus subtilis* group develops yellow color on the purple colored agar medium. Bromocresol purple is used in this media as a pH indicator to detect mannitol fermentation.

Supplementary Table T1. Biochemical tests for primary identification (identification flow chart of Bergey's manual of determinative bacteriology was followed for biochemical tests)

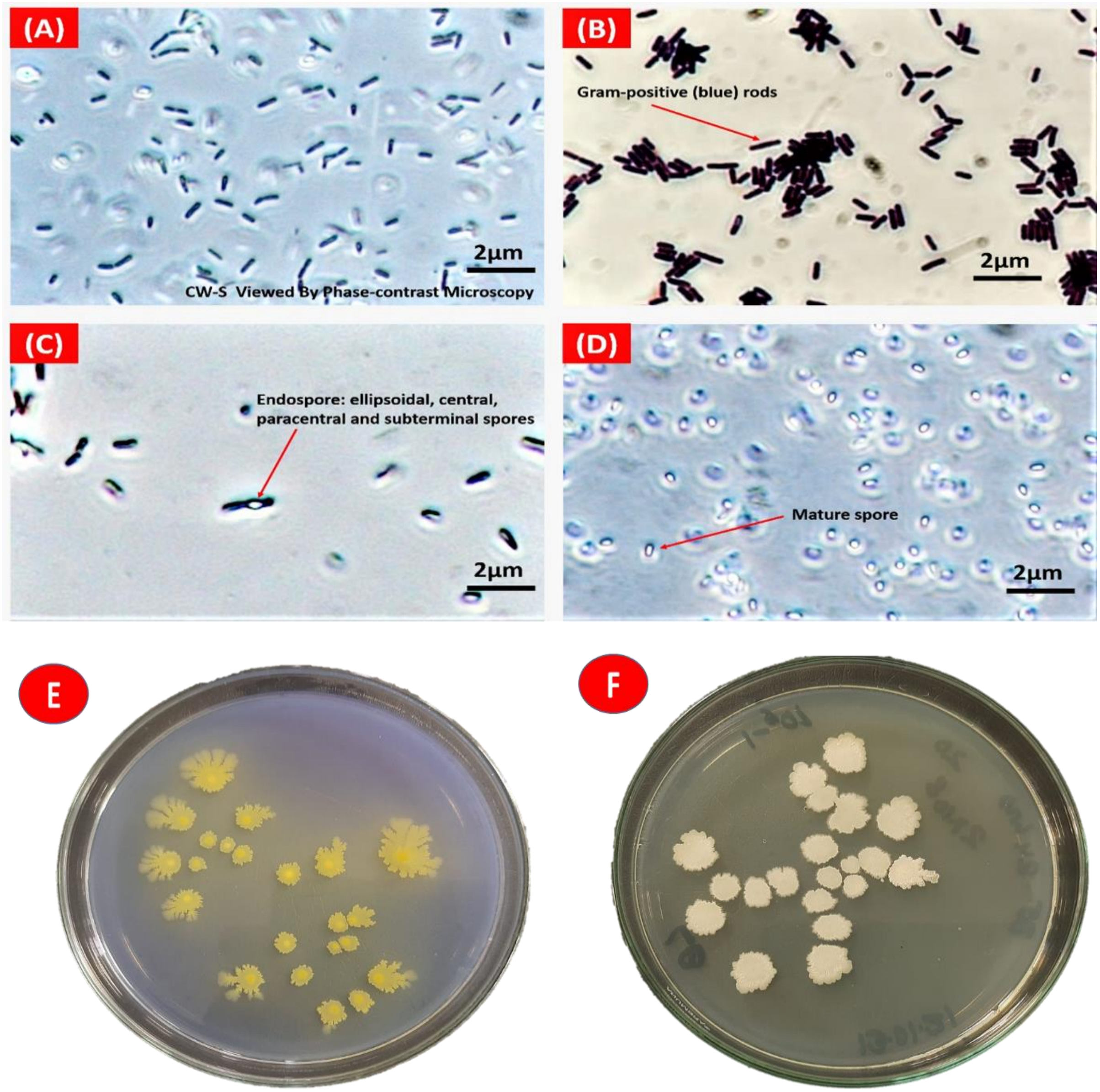

Supplementary Fig. F1. A) Shape of the CW-S cell under a phase-contrast microscope (Axio Imager A1, Carl Zeiss, Germany). **B)** The shape, arrangement, and gram reaction of a bacterial culture in a smear stained with the Gram Stain protocol demonstrate gram-positive results. **C)** *Bacillus subtilis:* ellipsoidal, central, paracentral and subterminal spores, not swelling the sporangia. **D)** Mother cell lyses and releases the mature spore at the end of fermentation cycle. **E)** Colony on differential media. **F)** Colony on QC plate.

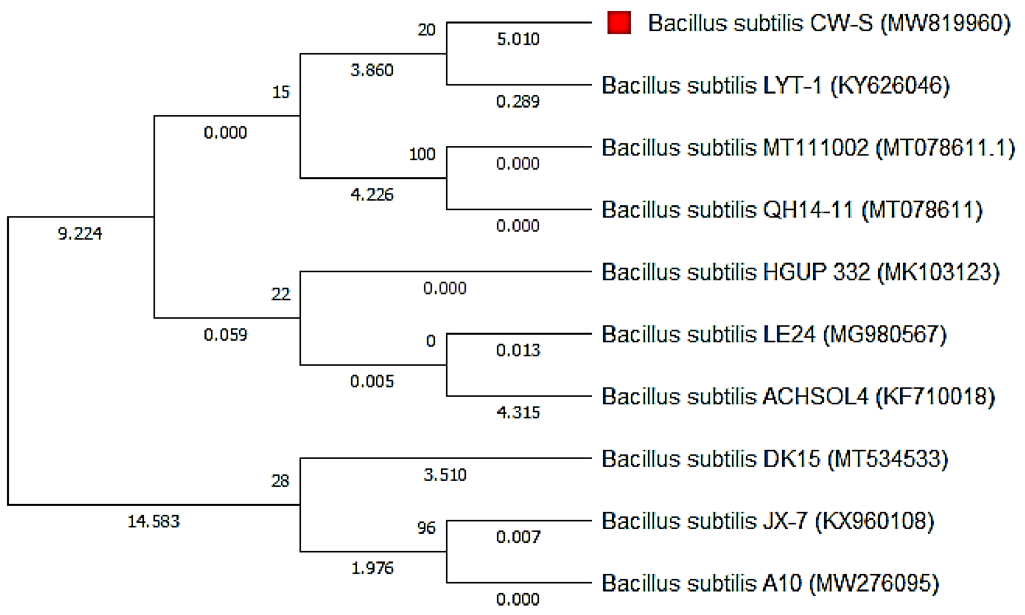

Supplementary Fig. F2. The evolutionary history was inferred by using the Maximum Likelihood method and Tamura-Nei model

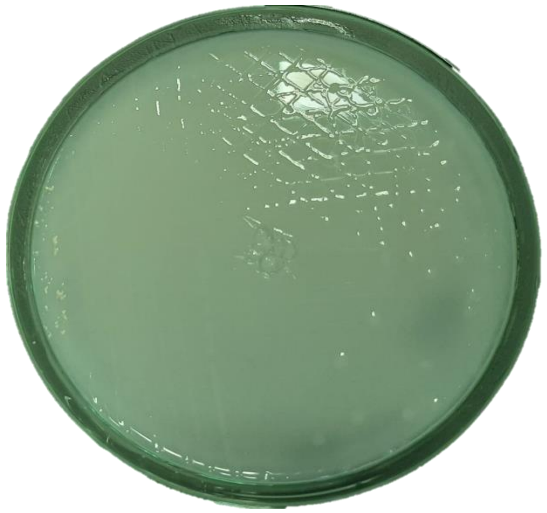

Supplemental Fig. F3. CW-S colony growth on nitrogen-free media

Run	Space type	Factor 1	Factor 2	Factor 3	Factor 4	Response
		**A:Temperature**	**B:Incubation Time**	**C:pH**	**D:Agitation**	**Y:CFU/mL (10^8)**
1	Factorial	-1	-1	1	-1	6.06 ± 0.18
2	Factorial	1	-1	-1	1	7.10 ± 0.11
3	Factorial	-1	1	1	1	6.16 ± 0.04
4	Factorial	-1	1	-1	-1	7.56 ± 0.08
5	Factorial	-1	-1	-1	1	6.64 ± 0.13
6	Factorial	1	-1	-1	-1	6.32 ± 0.09
7	Axial	0	-2	0	0	8.51 ± 0.07
8	Axial	0	0	0	-2	5.95 ± 0.12
9	Center	0	0	0	0	9.89 ± 0.03
10	Center	0	0	0	0	9.84 ± 0.17
11	Factorial	1	1	1	1	6.21 ± 0.06
12	Axial	0	0	-2	0	8.34 ± 0.11
13	Factorial	1	1	1	-1	5.95 ± 0.05
14	Factorial	-1	1	1	-1	6.87 ± 0.02
15	Factorial	1	1	-1	-1	6.81 ± 0.08
16	Center	0	0	0	0	9.75 ± 0.11
17	Axial	0	0	2	0	7.31 ± 0.10
18	Axial	-0	0	0	0	3.74 ± 0.13
19	Factorial	-1	1	-1	1	6.03 ± 0.05
20	Center	0	0	0	0	9.66 ± 0.19
21	Axial	2	0	0	0	3.18 ± 0.05
22	Axial	0	0	0	2	5.64 ± 0.14
23	Center	0	0	0	0	9.81 ± 0.17
24	Factorial	1	-1	1	-1	5.14 ± 0.15
25	Factorial	-1	-1	1	1	6.14 ± 0.09
26	Factorial	1	1	-1	1	6.69 ± 0.05
27	Axial	0	-2	0	0	8.90 ± 0.11
28	Factorial	-1	-1	-1	-1	7.20 ± 0.03
29	Factorial	1	-1	1	1	6.01 ± 0.11
30	Center	0	0	0	0	9.70 ± 0.16
Within columns, CFU ± SD values (n=3)						

Supplementary Table T2. Observed response of CCD using four independent variables and six centre points.

**Source**	**Std. Dev.**	**R^2**	**Adjusted R^2**	**Predicted R^2**	**PRESS**	
Linear	1.89	0.0374	-0.1166	-0.2758	117.90	
2FI	2.13	0.0652	-0.4269	-0.5484	143.10	
**Quadratic**	**0.1105**	**0.9980**	**0.9962**	**0.9904**	**0.8898**	**Suggested**
Cubic	0.0758	0.9996	0.9982	0.9962	0.3513	Aliased
Focus on the model maximizing the Adjusted R^2 and the Predicted R^2						

Supplementary Table T3. Model (CCD) summary statistics

**Source**	**Sum of Squares**	**df**	**Mean Square**	**F-value**	**p-value**	
Linear	88.92	20	4.45	582.70	< 0.0001	
2FI	86.35	14	6.17	808.41	< 0.0001	
**Quadratic**	**0.1449**	**10**	**0.0145**	**1.90**	**0.2481**	**Suggested**
Cubic	0.0021	2	0.0010	0.1349	0.8769	Aliased
Pure Error	0.0382	5	0.0076			
The selected model should have insignificant lack-of-fit.						

Supplementary Table T4. Lack of Fit Tests.

Run Order	Actual Value	Predicted Value	Residual	Leverage	Internally Studentized Residuals	Externally Studentized Residuals	Cook's Distance	Influence on Fitted Value DFFITS	Standard Order
1	6.06	6.17	-0.1096	0.583	-1.537	-1.617	0.220	-1.914	5
2	7.10	7.00	0.1033	0.583	1.449	1.509	0.196	1.786	10
3	6.16	6.11	0.0504	0.583	0.707	0.695	0.047	0.822	15
4	7.56	7.44	0.1204	0.583	1.689	1.813	0.266	2.145[1]	3
5	6.64	6.62	0.0237	0.583	0.333	0.323	0.010	0.382	9
6	6.32	6.39	-0.0746	0.583	-1.046	-1.049	0.102	-1.242	2
7	8.51	8.49	0.0196	0.583	0.275	0.266	0.007	0.315	19
8	5.95	5.91	0.0362	0.583	0.508	0.495	0.024	0.586	23
9	9.89	9.78	0.1150	0.167	1.140	1.153	0.017	0.515	28
10	9.84	9.78	0.0650	0.167	0.644	0.631	0.006	0.282	27
11	6.21	6.27	-0.0550	0.583	-0.771	-0.760	0.056	-0.900	16
12	8.34	8.47	-0.1304	0.583	-1.829	-2.004	0.312	-2.371[1]	21
13	5.95	6.00	-0.0479	0.583	-0.672	-0.659	0.042	-0.780	8
14	6.87	6.97	-0.1000	0.583	-1.402	-1.453	0.184	-1.720	7
15	6.81	6.82	-0.0100	0.583	-0.140	-0.136	0.002	-0.160	4
16	9.75	9.78	-0.0250	0.167	-0.248	-0.240	0.001	-0.107	26
17	7.31	7.16	0.1513	0.583	2.121	2.449	0.420	2.897[1]	22
18	3.74	3.75	-0.0054	0.583	-0.076	-0.073	0.001	-0.087	17
19	6.03	6.10	-0.0667	0.583	-0.935	-0.931	0.082	-1.101	11
20	9.66	9.78	-0.1150	0.167	-1.140	-1.153	0.017	-0.515	30
21	3.18	3.15	0.0262	0.583	0.368	0.357	0.013	0.423	18
22	5.64	5.66	-0.0154	0.583	-0.216	-0.209	0.004	-0.247	24
23	9.81	9.78	0.0350	0.167	0.347	0.337	0.002	0.151	25
24	5.14	5.07	0.0700	0.583	0.982	0.980	0.090	1.160	6
25	6.14	6.13	0.0133	0.583	0.187	0.181	0.003	0.214	13
26	6.69	6.60	0.0854	0.583	1.198	1.217	0.134	1.440	12
27	8.90	8.90	0.0013	0.583	0.018	0.017	0.000	0.020	20
28	7.20	7.14	0.0583	0.583	0.818	0.808	0.062	0.957	1
29	6.01	6.15	-0.1446	0.583	-2.027	-2.299	0.384	-2.720[1]	14
30	9.70	9.78	-0.0750	0.167	-0.744	-0.732	0.007	-0.327	29

[1] Exceeds limits.

Supplementary Table T5. Actual and Predicted Value of CCD

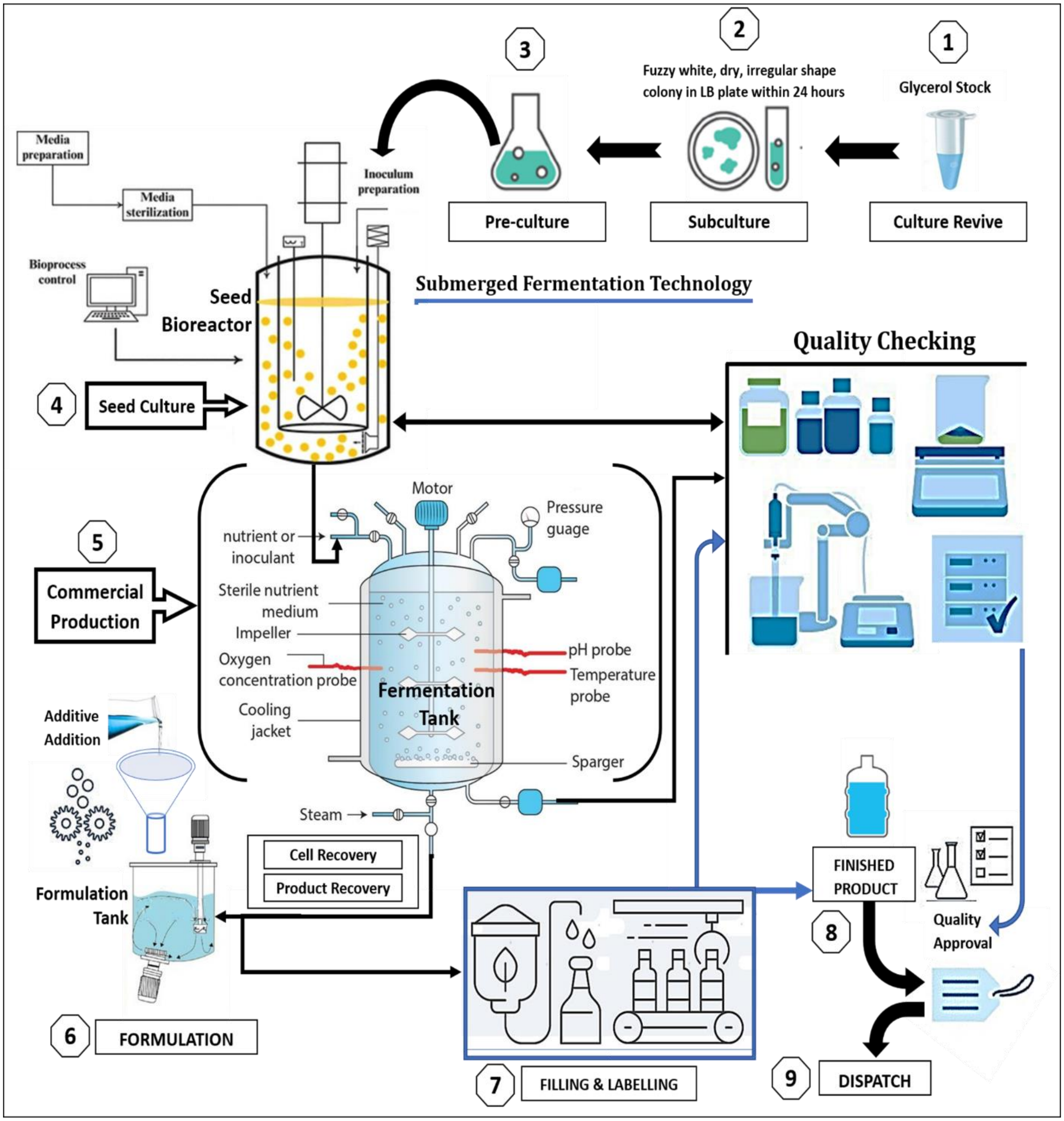

Supplementary Fig. F4. Industrial production process flow diagram of CW-S.

Month	Control : CFU/mL (10^9)	Formulated: CFU/mL (10^9)	Control: pH	Formulated: pH
0	2.010±0.0400a	1.97±0.040a	6.93±0.04a	6.86±0.057a
1	0.789±0.0355b	1.93±0.060a	6.55±0.20ab	6.79±0.057a
2	0.28±0.0488c	1.45±0.080b	6.19±0.11bc	6.47±0.047bc
3	0.125±0.0175d	0.795±0.013c	6.03±0.03cd	6.44±0.085c
4	0.0435±0.0035e	0.51±0.020d	5.70±0.09d	6.51±0.071bc
5	0.00078±6.03E-05 e	0.44±0.052d	5.16±0.05e	6.71±0.165ab
6	3.23E-06±5.51E-07e	0.38±0.029d	4.57±0.31f	6.66±0.057abc
* Within columns, mean ± SD values with a shared letter do not differ significantly (P < 0.05), according to Tukey multiple comparisons of means test (n=3)				

Supplementary Table T6. Cell concentration (CFU/mL) viability and pH variability of control and formulated CW-S with time interval.

Nutrient adding	Initial cell concentration of CW-S (CFU/mL) after mixing with nutrient solution	Cell concentration of CW-S (CFU/mL) at seventh days
cycle1	$2.56±0.07×10^6$	$5.62±0.12×10^3$
cycle2	$2.18±0.05×10^6$	$3.88±0.09×10^4$
cycle3	$2.68±0.13×10^6$	$4.16±0.05×10^4$
cycle4	$1.57±0.14×10^6$	$5.19±0.17×10^3$
cycle5	$3.84±0.09×10^6$	$8.54±0.13×10^3$
cycle6	$2.55±0.16×10^6$	$2.98±0.02×10^4$
cycle7	$3.16±0.11×10^6$	$6.24±0.04×10^4$
cycle8	$2.33±0.02×10^6$	$1.28±0.18×10^4$
cycle9	$1.98±0.08×10^6$	$5.84±0.24×10^4$
cycle10	$2.64±0.03×10^6$	$3.42±0.17×10^4$

cycle11	$3.12 \pm 0.03 \times 10^6$	$6.57 \pm 0.34 \times 10^4$
CFU mean ± SD values (n=3)		

Supplementary Table T7. Cell concentration of CW-S in nutrient solution of aeroponic

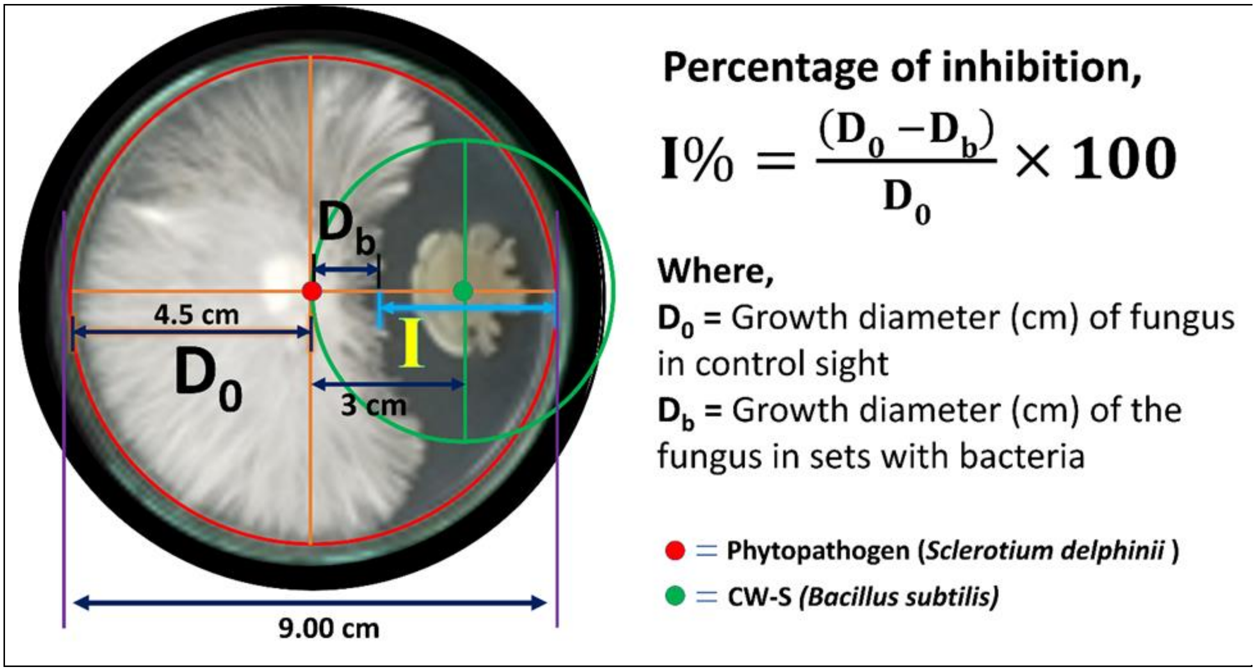

Supplementary Fig. F5. Schematic representation of inhibition percentage. The biocontrol activity assay was carried out in petri plates (9 cm in diameter) with ten replications, and the results were expressed as the mean of these values.

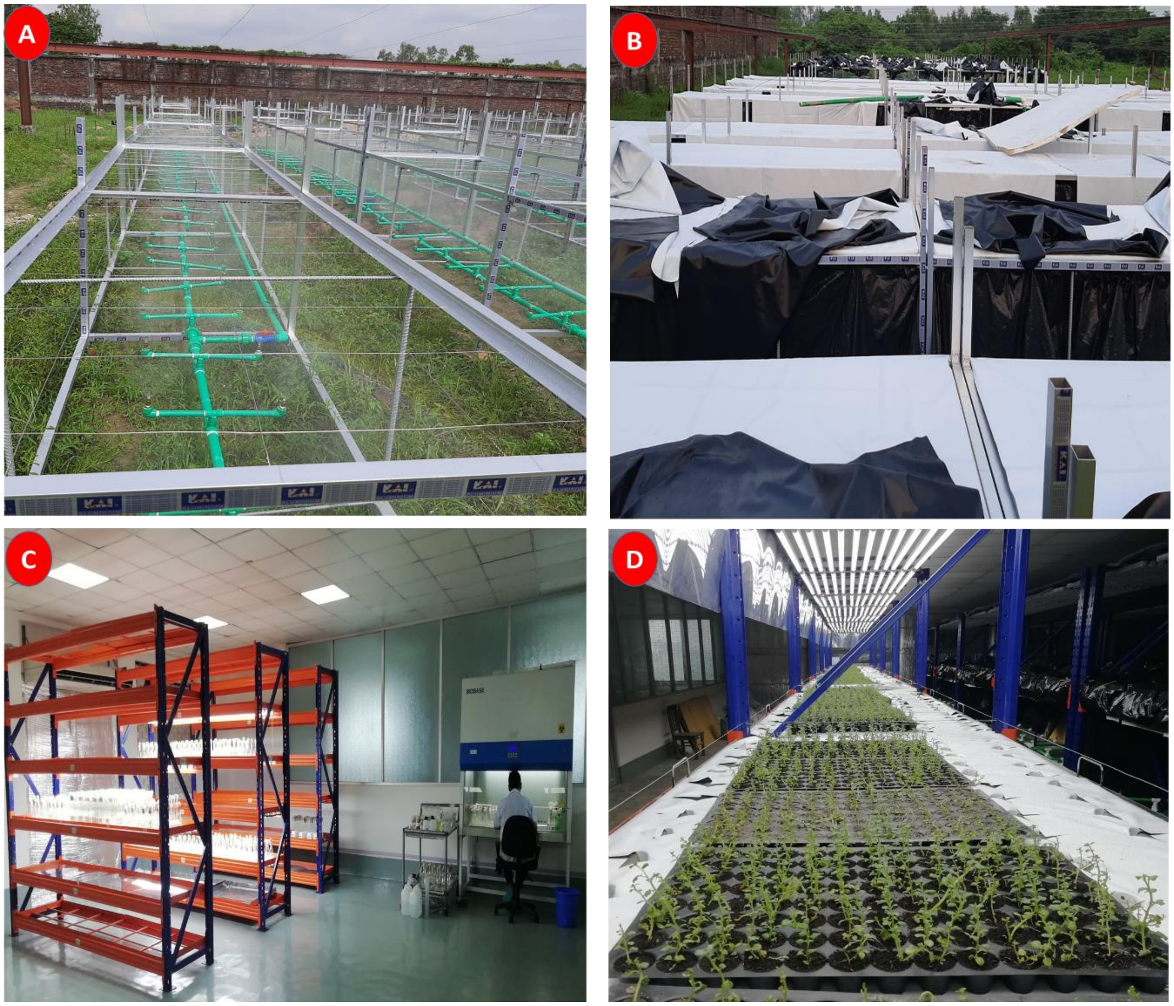

Supplementary Fig. F6. A&B) Aeroponic rack installation. **C)** Tissue culture plantlets production. **D)** Hardening of plantlets.

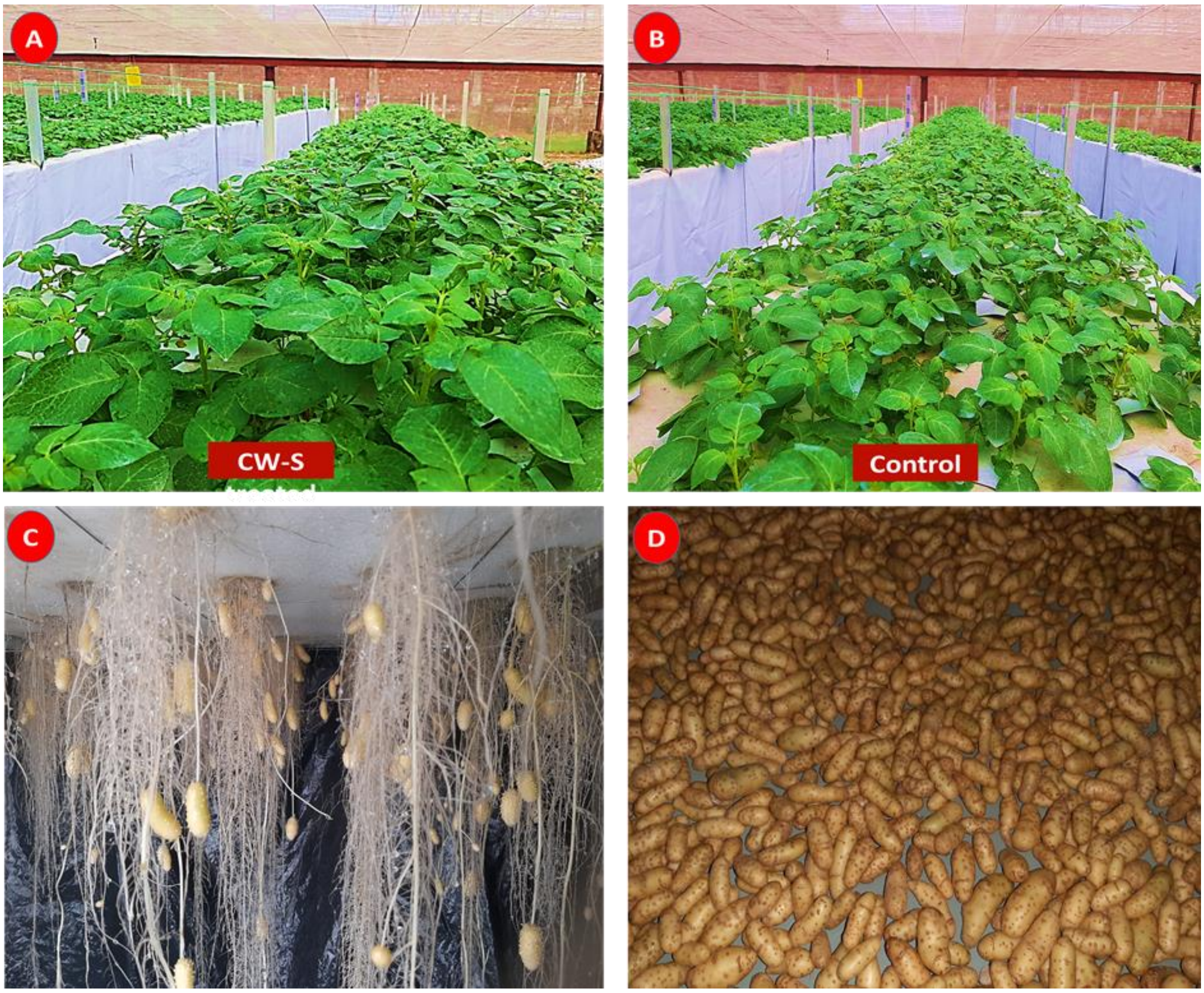

Supplementary Fig. F7. A) Growth of treated plantlets in aeroponic. **B)** Growth of control plantlets in aeroponic. . **C)** Minituber initiation. **D)** Harvested minituber.

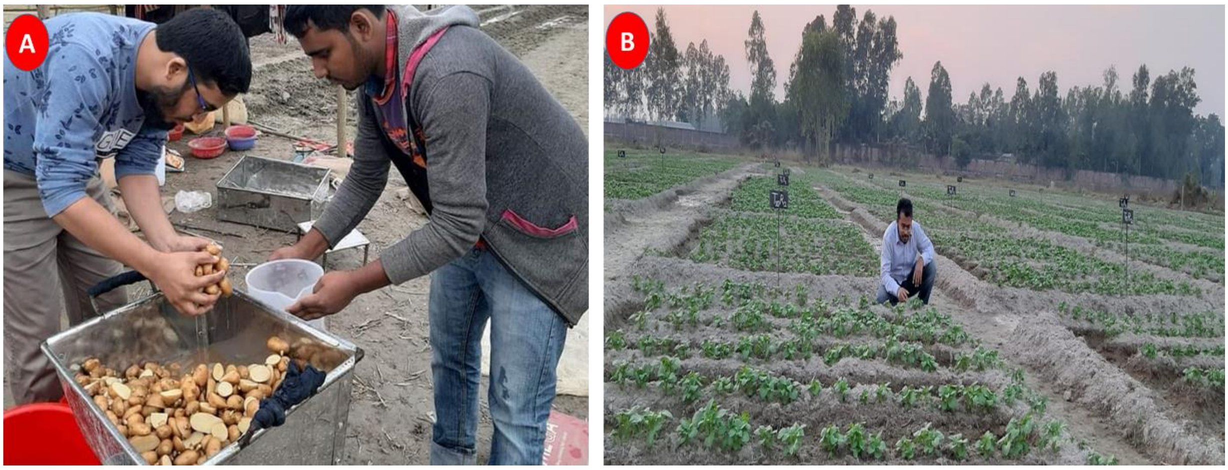

Supplementary Fig. F8. **A)** In a field trial, tuber treatment with CW-S. **B)** Field condition after 15 days of germination.

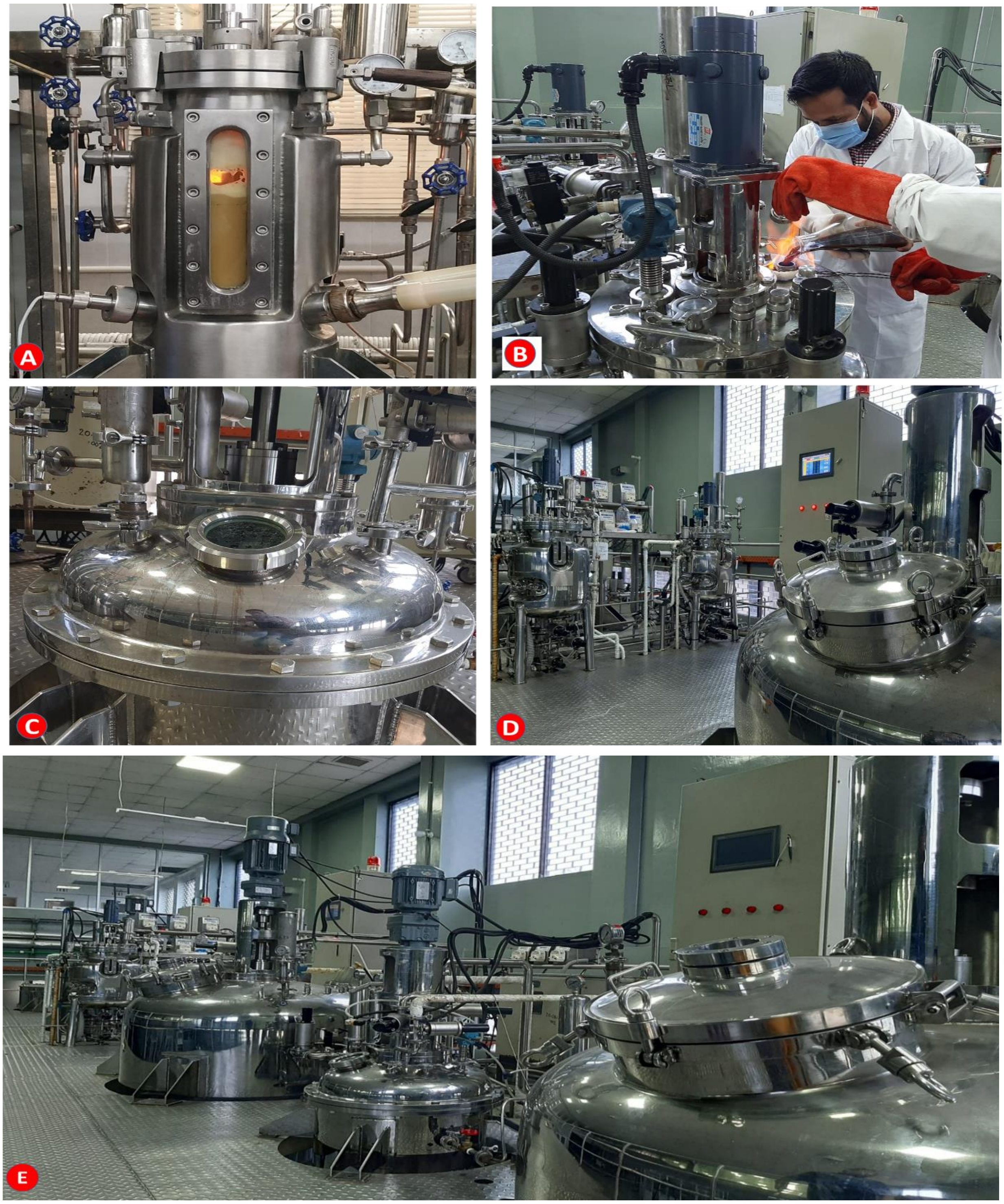

Supplementary Fig. F9: **A**) Laboratory Bioreactor: 10L. **B**) Seed culture vessel: 30L. **C**) Pilot Bioreactor: 300L. D. Industrial Bioreactor: 3000L. E) Fermentation floor.
